# Allele-Specific Down-Regulation of *RPTOR* Expression Induced by Retinoids Contributes to Climate Adaptations

**DOI:** 10.1371/journal.pgen.1001178

**Published:** 2010-10-28

**Authors:** Chang Sun, Catherine Southard, David B. Witonsky, Ralf Kittler, Anna Di Rienzo

**Affiliations:** Department of Human Genetics, University of Chicago, Chicago, Illinois, United States of America; University of Washington, United States of America

## Abstract

The mechanistic target of rapamycin (MTOR) pathway regulates cell growth, energy homeostasis, apoptosis, and immune response. The regulatory associated protein of MTOR encoded by the *RPTOR* gene is a key component of this pathway. A previous survey of candidate genes found that *RPTOR* contains multiple SNPs with strong correlations between allele frequencies and climate variables, consistent with the action of selective pressures that vary across environments. Using data from a recent genome scan for selection signals, we honed in on a SNP (rs11868112) 26 kb upstream to the transcription start site of *RPTOR* that exhibits the strongest association with temperature variables. Transcription factor motif scanning and mining of recently mapped transcription factor binding sites identified a binding site for POU class 2 homeobox 1 (POU2F1) spanning the SNP and an adjacent retinoid acid receptor (RAR) binding site. Using expression quantification, chromatin immunoprecipitation (ChIP), and reporter gene assays, we demonstrate that POU2F1 and RARA do bind upstream of the *RPTOR* gene to regulate its expression in response to retinoids; this regulation is affected by the allele status at rs11868112 with the derived allele resulting in lower expression levels. We propose a model in which the derived allele influences thermogenesis or immune response by altering MTOR pathway activity and thereby increasing fitness in colder climates. Our results show that signatures of genetic adaptations can identify variants with functional effects, consistent with the idea that selection signals may be used for SNP annotation.

## Author Summary

Climate has exerted strong selective pressures in human populations during their dispersal, and signatures of these adaptations are still detectable in the geographic distribution of polymorphisms. *RPTOR* is a key component of the mechanistic target of rapamycin pathway, which regulates cell growth, metabolism, and immune response; and its deregulation is associated with human diseases, including cancer and diabetes. Previous studies showed that variation in *RPTOR* carry strong signatures of adaptations to different climates. Here, we used evolutionary genetics approaches coupled with transcription factor motif data mining to refine the location of the selection target. We then used functional assays to show that the selected polymorphism resides in a sequence element that regulates gene expression levels in response to retinoids. The derived allele at this SNP, which results in lower expression levels, increases in frequency with decreasing temperatures, consistent with the notion that it confers a selective advantage in colder climates possibly through its effects on energy metabolism or immune response. These results suggest a novel regulatory role for retinoids in MTOR signaling. Moreover, they support the proposal that evolutionary approaches can be informative for SNP functional annotation.

## Introduction

A major goal of human genetics is to identify functional genomic regions, especially those containing variants that influence common disease susceptibility or disease-related phenotypes. However, due to the complexity of the genome, it is not easy to distinguish functional from non-functional regions, especially for regulatory elements, which can lie far from the target gene. Because adaptive variation must necessarily have functional, in addition to fitness effects, signals of positive natural selection have been proposed as an informative approach to the functional annotation of the genome. Many genome-wide selection scans have been performed to date based on different approaches (as reviewed by references [1]–[5]). These studies have generated a large number of signals, most of which await validation through functional or phenotypic analyses.

One approach to the detection of local adaptations searches for correlations between allele frequencies and environmental variables, e.g. latitude or temperature; this approach assumes that the intensity of selection varies across environments and that the variables correlated with allele frequencies are good proxies for the true selective pressure (e.g. temperature is a proxy for cold or heat stress). This approach may be particularly informative for human populations who originated in Sub-Saharan Africa and migrated out of Africa 60–100k years ago to occupy most of the earth landmass [6], [7]. During this dispersal, human populations have been exposed to extremely diverse environments, which differ in terms of climate, including temperature, day length, UV radiation, pathogen diversity and other factors. These aspects of human environments have important effects on physiological and developmental processes and, therefore, exerted strong selective pressures on the human genome [8]. Consistent with the action of spatially-varying selective pressures, human skin pigmentation [9], body mass [10], basal metabolic rates (BMR) [11], and cranial form [12] vary across human populations and are associated with climate variables. It was recently shown that polymorphisms in candidate genes for metabolic disorders [13], salt homeostasis [14], [15], response to stress [16], [17], and circadian signaling [18], are strongly correlated with climate variables, thus providing a possible genetic mechanism for the observed distribution of human phenotypes across populations.

One of these studies identified the *RPTOR* gene as a target of spatially-varying selective pressures because many variants within the gene exhibited particularly strong correlations between allele frequency and latitude [13]. The *RPTOR* gene codes for a protein involved in the target of rapamycin (MTOR) pathway, which in turn is important in cell growth, proliferation, apoptosis [19], and immune response [20]. Two multiprotein complexes, MTORC1 and MTORC2, constitute the core of this pathway [19]; MTORC1 is the target of and sensitive to rapamycin, an immunosuppressant and anti-cancer agent, while the other complex is not [19]. Under the regulation of nutrient, energy, and stress, MTORC1 can transfer the proliferation signal to the downstream proteins mainly by phosphorylating two substrates, ribosomal protein S6 kinase, 70kDa, polypeptide 1 (RPS6KB1) and eukaryotic translation initiation factor 4E binding protein 1 (EIF4EBP1) [19]. The regulatory associated protein of MTOR (RPTOR) is a crucial component of the MTORC1 [21], which works both as a scaffold and a regulatory protein [21], [22]. In particular, RPTOR can bind to TOR signaling (TOS) domain of EIF4EBP1 and RPS6KB1 [23]– and to the HEAT repeat domain of MTOR [21], thus making the phosphorylation reaction possible. Therefore, in absence of RPTOR, the kinase activity of MTOR is mainly reduced or inhibited [21]. Given the function of this pathway and its regulation in response to environmental stimuli, it is plausible that the *RPTOR* variants correlated with latitude, or one in strong linkage disequilibrium (LD) with these SNPs, conferred adaptations to selective pressures that vary across environments. However, the mechanism through which this variation affects the function of the *RPTOR* gene remains unknown.

In the present study, we used population genetics analyses and *in vitro* functional assays to localize the most likely target of selection and to propose a mechanism underlying its effect on *RPTOR* gene function. More specifically, we used the results of a genome-wide selection scan to identify the variant with the strongest evidence as a target of climate adaptations (Hancock and Di Rienzo, personal communication). This SNP lies within a predicted POU class 2 homeobox 1 (POU2F1) binding site and near a retinoid acid receptor (RAR) binding site identified by Chromatin immunoprecipitation (ChIP)-chip [26]. Given that POU2F1 and RAR are known to cooperate in the regulation of gene expression, we hypothesized that this SNP is located within an enhancer that regulates *RPTOR* expression in response to retinoid acid (RA). Consistent with this prediction, we observed a significant increase of *RPTOR* expression in both MCF-7 and HepG2 cell lines after treatment with RA. We further showed that both POU2F1 and RAR bind to the region spanning the SNP of interest in both cell lines. Finally, we determined that the two alleles at this SNP influence RA-mediated transcriptional response by means of reporter gene assays using enhancer constructs containing, respectively, the ancestral and the derived allele. Based on these results, we propose that the SNP that is strongly correlated with climate variables affects fitness by influencing *RPTOR* gene expression.

## Results

### Refining the location of the selection target

**Figure 1:**
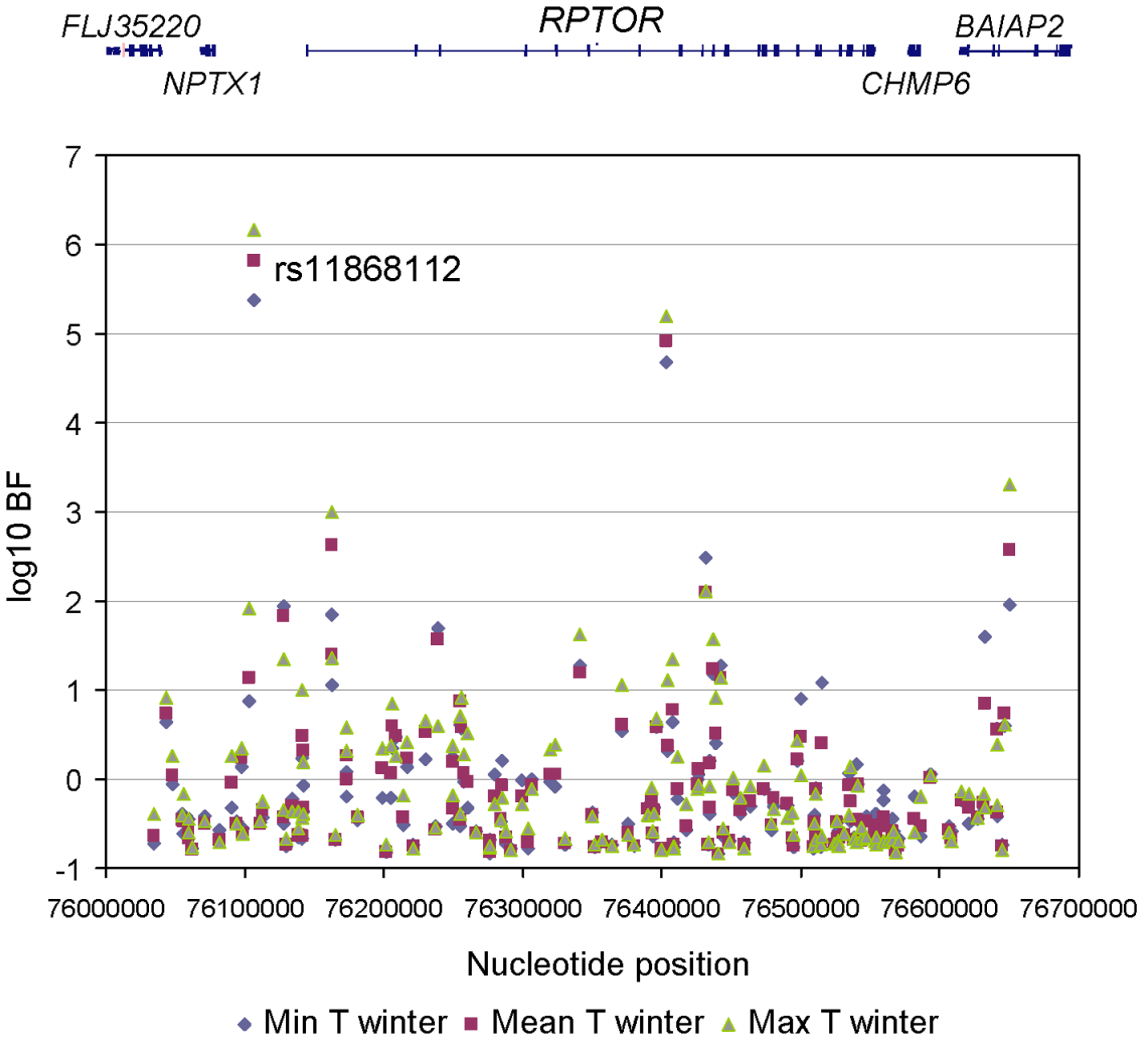
Association between 156 *RPTOR* SNPs and winter temperature variables. The nucleotide position of each SNP (based on build 36) is shown on the horizontal axis and the log_10_ BF is shown on the vertical axis. The RefSeq genes in this region are displayed at the top.

**Figure 2:**
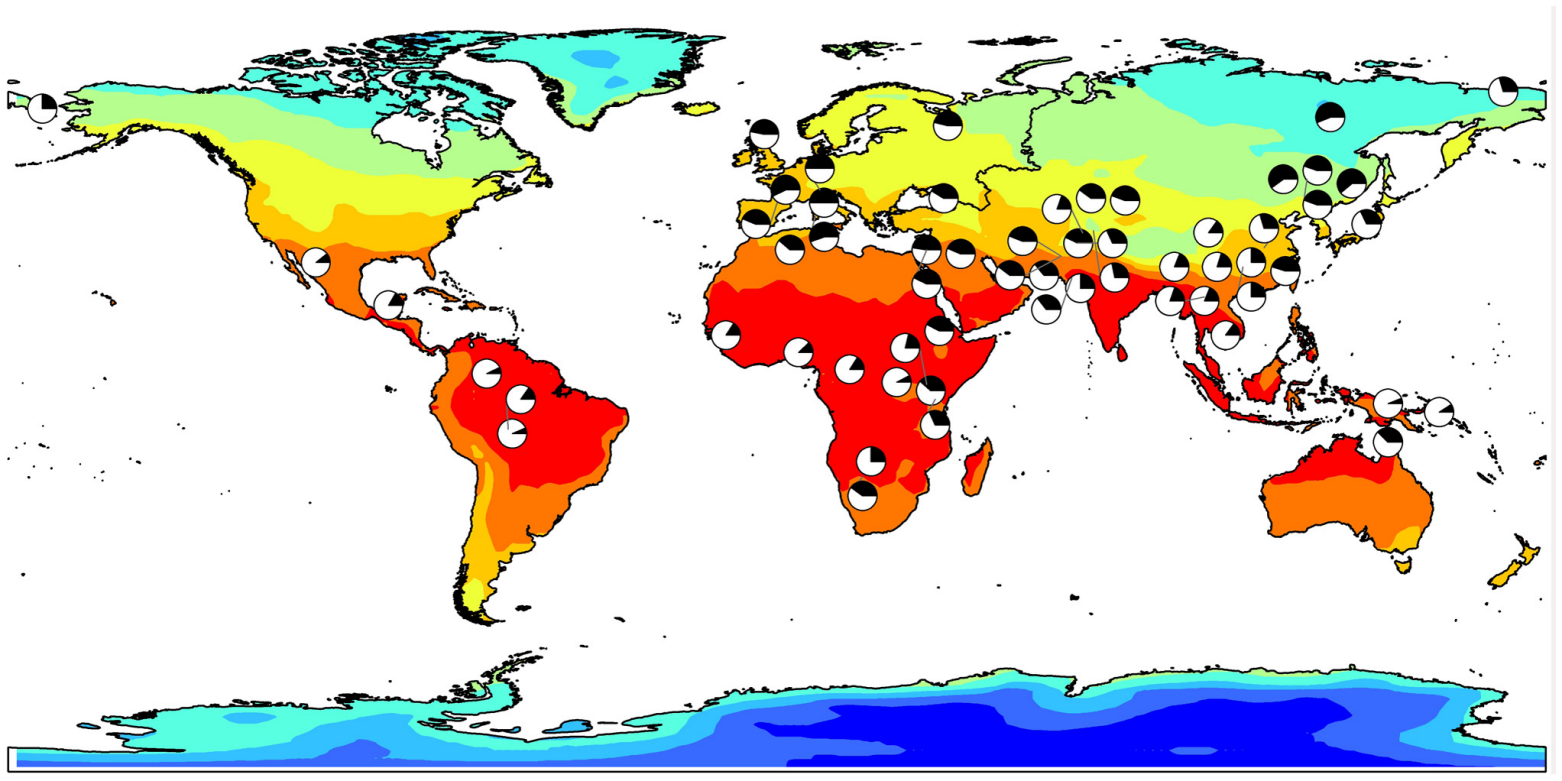
Allele frequency for rs11868112 (ancestral and derived alleles are shown in white and black, respectively) in HGDP populations mapped onto a GIS map of Winter maximum temperature.

To refine the location of the polymorphism targeted by climate-related selective pressures, we mined the results of a recent genome-wide scan for signals of allele frequency correlation with climate variables (Hancock and Di Rienzo, personal communication). Allele frequencies for a total of 156 SNPs were obtained in the genomic region spanning the *RPTOR* gene and 100 kb upstream and downstream of the gene (See Table S1 for detailed information). The evidence for the action of selective pressures related to climate was assessed by means of a Bayesian method that yields a Bayes factor (BF), which is a measure of the support for a model in which a SNP allele frequency distribution is linearly dependent on a climate variable in addition to population structure, relative to a model in which the allele frequency distribution is dependent on population structure alone [27]. A transformed rank statistic (sometime referred to as an ‘empirical *p*-value’) was also calculated to determine whether the BF value of a SNP of interest is unusual relative to those of other SNPs matched by allele frequency; as with formal *p*-values, a low rank indicates strong evidence for a correlation (i.e. a large BF) [27]. We examined the following climate variables: mean, minimum and maximum temperature, precipitation rate, relative humidity and solar radiation; for all climate variables we considered the value in the winter and summer, respectively. Among the 156 SNPs tested, 56 had a rank lower that 5% for at least one climate variable. One SNP, rs11868112, had particularly large BFs with winter temperatures (rank statistic = 0.0082, 0.0064, and 0.0039 for minimum, mean and maximum winter temperature, respectively. See Figure 1 and Figure 2, Table S2 and Table S3for detail.). Therefore, this SNP, or one in strong LD with it, is a candidate target of selective pressures related to climate.

To determine whether an unknown coding SNP could account for the above correlations with climate variables, we re-sequenced the *RPTOR* cDNA from a small, but diverse group of HapMap lymphoblastoid cell lines. As shown in Figure S1, 38 SNPs were identified; 5 and 17 of them were located in 5′ and 3′ untranslated regions, respectively. No non-synonymous SNP was identified. Moreover, all of them showed a relatively low LD with rs11868112 (*r* ^2^<0.62, 0.34, and 0.69 in YRI, CEU, and ASN populations, respectively). These results indicate that the selection signal is not due to a non-synonymous SNP and suggest that the selection target is not likely to be within the coding region. Because the true target of selection could be a regulatory SNP, we re-sequenced also 22.6kb of conserved non-coding elements near the *RPTOR* gene, the *RPTOR* promoter, and a 2.6 kb region spanning rs11868112. As shown in Figure S2, 11, 16, and 135 SNPs were found in the intergenic, promoter, and intron regions, respectively; polymorphism levels were within the range of genome-wide variation (see Table S4) [28], [29]. Moreover, none of the additional 161 SNPs discovered in this survey showed strong LD with rs11868112 (*r* ^2^<0.23, 0.48, and 0.54 in YRI, CEU, and ASN populations, respectively, result not shown). Therefore, our targeted re-sequencing survey did not identify SNPs with likely functional effects and that could drive the signal seen at rs11868112.

We used the re-sequencing data to perform neutrality tests based on the allele frequency spectrum, but no significant departure was detected (Table S4). This may be due to the fact that these tests are known to have inadequate power under a range of selection scenarios, including models in which selection acted on an allele occurring at appreciable frequencies prior to the onset of selection [30]–[32].

SNP rs11868112 lies 26.2 kb 5′ to the *RPTOR* gene, which is ubiquitously expressed and is a strong biological candidate for adaptations to different local environments, and 41.3 kb 3′ to the *NPTX1* gene, which is transcribed in the opposite orientation relative to *RPTOR*. *NPTX1* codes for neuronal pentraxin 1 that is expressed only in the central neurons of the nervous system where it plays a role in synaptic plasticity [33]; given its function, the *NPTX1* gene is a less likely target of adaptations to different climates. We hypothesized that the SNP rs11868112 is located within a long-distance regulatory element and that this SNP influences the activity of this regulatory element. This hypothesis was bolstered by the fact that this SNP lies less than 1 kb away from a retinoic acid receptor α (RARA) binding site detected by ChIP-chip in the breast cancer cell line MCF-7 [26]. We also found that rs11868112 resides within a canonical POU2F1 binding site, as predicted by the Match program in the TRANSFAC database (http://www.gene-regulation.com). Since POU2F1 is known to cooperate with RARA to regulate gene expression [34], we hypothesize that *RPTOR* gene expression is regulated by retinoids via activation of the RARA and that rs11868112 modulates the activation of *RPTOR* expression by modifying POU2F1 binding affinity to the DNA.

### Regulation of *RPTOR* expression by retinoids

**Figure 3:**
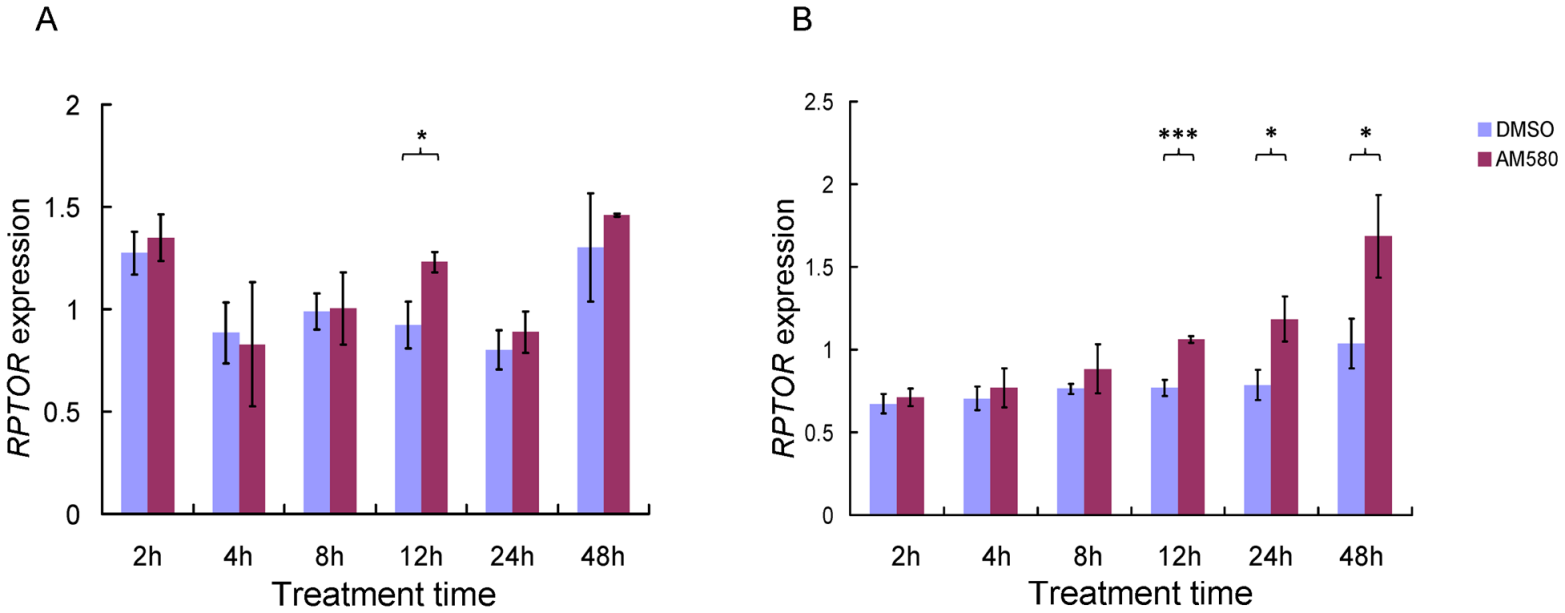
Effect of retinoids on *RPTOR* expression in HepG2 (A) and MCF-7 (B) cell lines at different treatment times. Each bar indicates the average of three independent biological replicates and the error bar denotes standard deviation. * *P*<0.05; *** *P*<0.001.

To investigate the effects of retinoids on *RPTOR* expression, we treated HepG2 and MCF-7 cell lines with the selective RARA agonist AM580, which has greater specificity for RARA compared to all-*trans* RA [35], for different time periods (2–48 hrs) and measured *RPTOR* mRNA levels by quantitative real time PCR. The MCF-7 cell line was included because the RARA binding site was originally identified in these cells while the human hepatocellular carcinoma cell line HepG2 was included because the liver plays a prominent role in biological processes relevant to energy metabolism (e.g. carbohydrate and lipid metabolism). The genotype of rs11868112 is TT and CT for MCF-7 and HepG2, respectively. As shown in Figure 3A, *RPTOR* expression in HepG2 varied substantially across time points for vehicle (DMSO) control treatment. A 33.7% higher expression level was observed for 12 hrs treatment with AM580 versus DMSO (*P* = 0.01). For all other time points, no significant difference (*P*>0.05) was observed for AM580 and DMSO treatment. In MCF-7 cells, where we observed considerably less variation in *RPTOR* expression for DMSO treatment across time points, we found a significant increase of *RPTOR* mRNA levels upon AM580 treatment at 12, 24 and 48 hrs (38.1%, 50.8%, and 62.5% higher expression, and *P*<0.001, *P* = 0.01, and *P* = 0.02 respectively, Figure 3B). A relatively late (12 hrs or more) transcriptional response to retinoids has been observed for many other target genes of RARA [34], [36], [37]; however, it remains unclear whether the *RPTOR* gene is a direct or indirect target. These findings suggest that *RPTOR* expression may be regulated by RARA binding to the genomic region adjacent to SNP rs11868112.

### Binding of RARA and POU2F1 to the rs11868112 region

**Figure 4:**
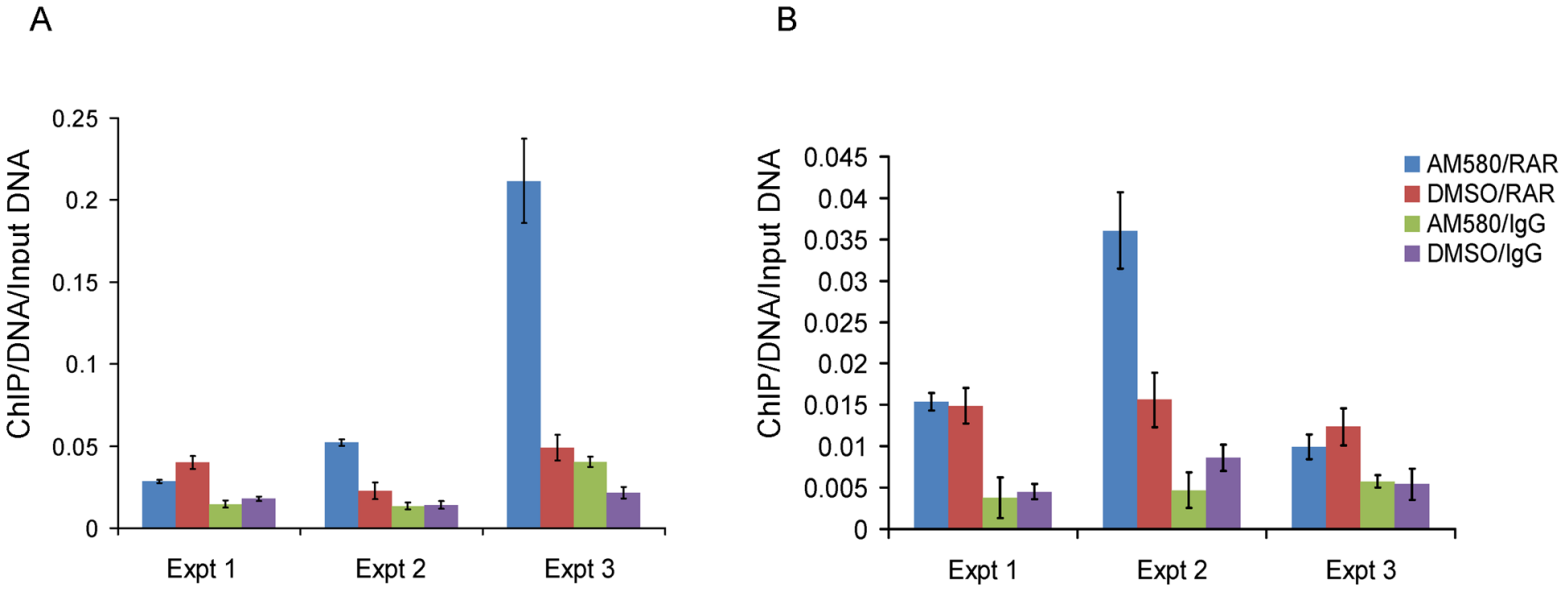
Enrichment of the region nearby rs11868112 in Anti-RAR ChIPed DNA relative to rabbit IgG ChIPed after AM580 and DMSO treatment. In HepG2 (A) and MCF-7 (B). Each bar indicates the average of three real time PCR technical replicates normalized by the input DNA and the error bar denotes standard deviation.

To investigate the role of SNP rs11868112 in the regulation of *RPTOR* expression, we performed ChIP assays followed by quantitative PCR to determine whether RARA and/or POU2F1 bind the DNA near the SNP. First, we treated HepG2 and MCF-7 cells with AM580 and DSMO and performed a ChIP with antibodies against RARA followed by real time PCR quantification of the region spanning the RARA binding site detected by ChIP-chip [26]. We found a significant enrichment (*P*<0.02) of the putative RARA binding region for the chromatin immunoprecipitated DNA with the RARA antibody (Figure 4A and 4B), which confirms RARA binding to the region near rs11868112. This enrichment was observed in both DMSO and AM580 treated cells (Figure 4A and 4B), which is consistent with the model for the genomic actions of retinoic acid receptors [38].

**Figure 5:**
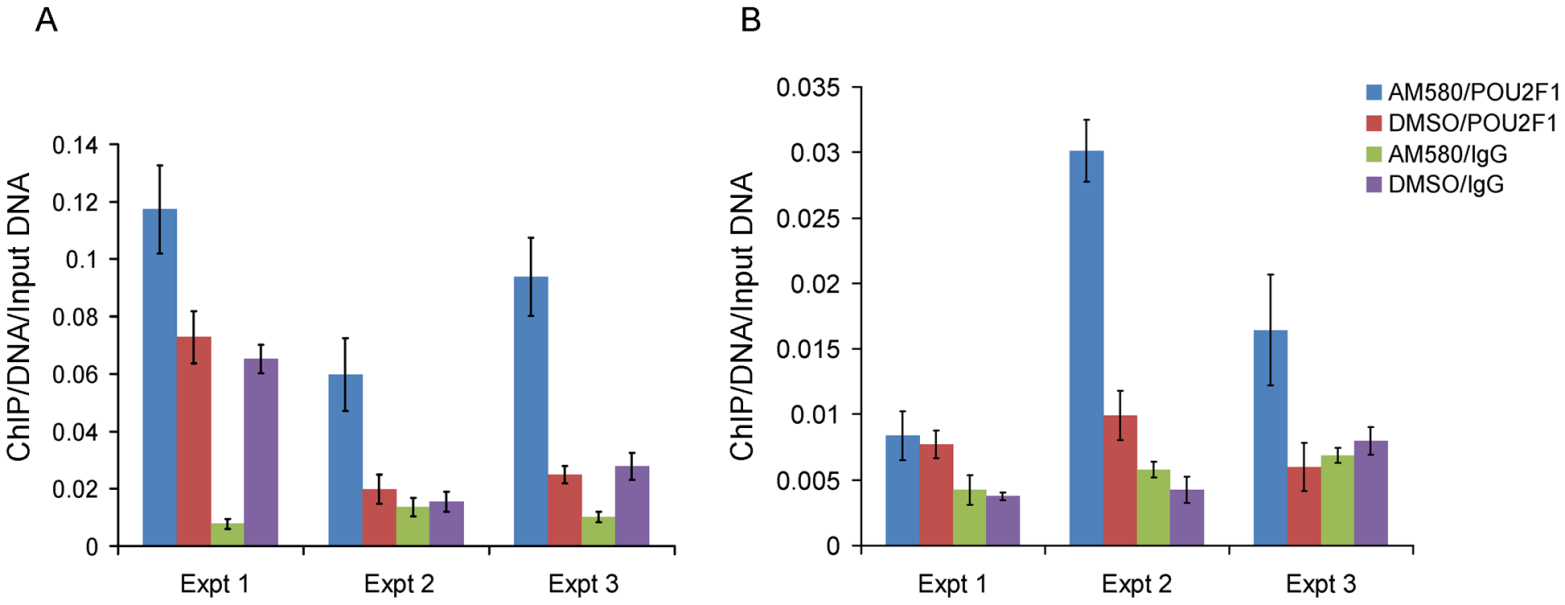
Enrichment of the region spanning rs11868112 in Anti-POU2F1 ChIPed DNA relative to rabbit IgG ChIPed after AM580 and DMSO treatment. In HepG2 (A) and MCF-7 (B). Each bar indicates the average of three real time PCR technical replicates normalized by the input DNA and the error bar denotes standard deviation.

To investigate whether POU2F1 binds to the region encompassing rs11868112 (in a canonical POU2F1 motif) and to study the retinoic acid dependency of POU2F1 recruitment, we performed ChIP with antibodies against POU2F1 in the same cell lines. Upon AM580 treatment, we observed a significant enrichment (*P*<0.01) of the putative POU2F1 binding region in the chromatin immunoprecipitated with the POU2F1 antibody in both HepG2 and MCF-7 cells (Figure 5A and 5B), which indicates POU2F1 binding to the genomic region encompassing rs11868112. For vehicle treatment we observed no significant POU2F1 binding in HepG2 cells (*P*>0.2, Figure 5A), but a significant enrichment in MCF-7 cells (Figure 5B), which suggests that POU2F1 binding to this specific genomic locus may not require liganded RARA dependent on the specific cell lineage. Overall, our findings indicate that RARA and POU2F1 bind to the region adjacent to and encompassing rs11868112, respectively, suggesting that this region acts as a *cis*-regulatory module with POU2F1 and RARA-binding elements.

### Enhancer activity of the rs11868112 alleles

**Figure 6:**
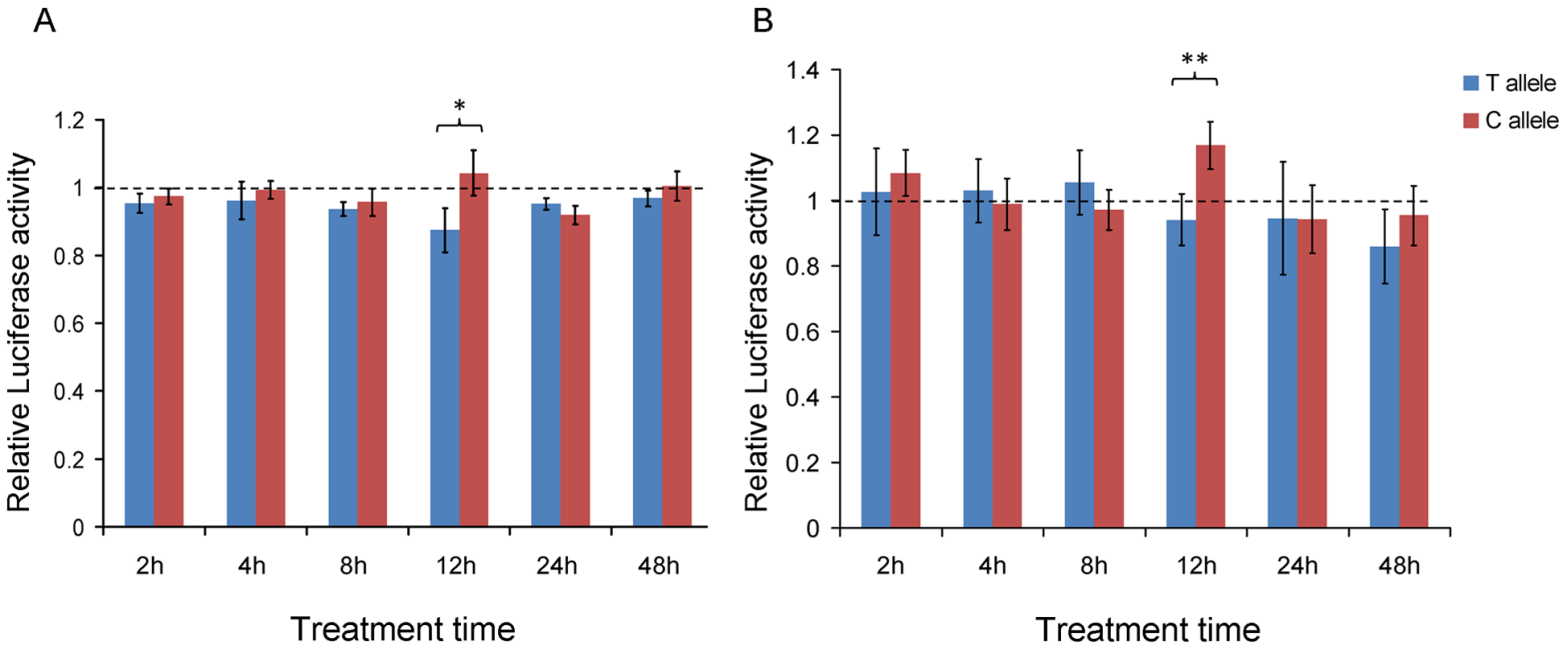
Reporter gene assays. In HepG2 (A) and MCF-7 (B) at different times after treatment with AM580. The relative luciferase activity is determined by the ratio between AM580 and DMSO treatment. Data is expressed as mean ± standard deviation. * *P*<0.05; ** *P*<0.01.

To test whether POU2F1 and RARA binding to this module elicits *cis*-regulatory effects dependent on the allele status of rs11868112, we performed luciferase reporter gene assays with the cloned regions of the ancestral and the derived allele. In HepG2 cells, the reporter gene construct containing the ancestral allele (C) exhibited a 19.2% higher luciferase activity than the construct for the derived allele (T) allele (*P* = 0.011) 12 hrs after AM580 treatment (Figure 6A). Before and after this time point, no significant difference was observed between the reporter gene constructs for the C and T alleles (*P*>0.09). This observation is consistent with the maximal RA-dependent induction of *RPTOR* expression at 12 hrs after AM580 treatment. Similar results were obtained with the MCF-7 cell line, where the reporter construct for the ancestral allele showed a 24.1% higher luciferase activity than those for the derived allele at 12 hrs after AM580 treatment (*P* = 0.0053, Figure 6B). These findings suggest that the region 26.2 kb upstream of *RPTOR* acts as an RA-dependent enhancer in human cells and that the activity of this enhancer depends on the allele status within the POU2F1 binding site at rs11868112.

## Discussion

In this study, we combined population genetics, bioinformatics, and experimental approaches to identify a *cis*-regulatory element harboring a SNP (rs11868112) associated with a strong signal of selection identified in a genome-wide study. The allele frequencies at this SNP are strongly correlated with latitude and winter temperature variables. A re-sequencing survey did not identify additional SNPs that are in strong LD with rs11868112 and that are likely to have functional effects. Because SNP rs11868112 lies within a predicted POU2F1 binding site located close to a RARA binding site identified by ChIP-chip, we hypothesized that this SNP influences the transcriptional response to RA. Consistent with this hypothesis, we showed that POU2F1 and RARA do bind to the genomic region spanning and near SNP rs11868112, respectively. Furthermore, reporter gene assays suggest that this region functions as a RA-dependent enhancer and that the allele status at rs11868112 affects enhancer activity. Although we cannot conclusively identify the target gene of this enhancer, *RPTOR* appears to be a strong candidate because it is induced by the selective RARA agonist AM580 in two different cell lines. The fact that the time of differential *RPTOR* expression in response to RA treatment coincides with the time of allele-specific down-regulation in reporter gene assays further supports this proposal. Overall, these results provide an example of how a selection signal can identify a functional SNP and suggest a role for the regulation of *RPTOR* expression in human adaptations to different environments.

Despite the clear signal of selection given by the correlation between allele frequency and climate variables, standard neutrality tests did not detect a significant departure from expectations. This included tests of the frequency spectrum as well as haplotype homozygosity (as assessed by the extended haplotype homozygosity [39] or integrated haplotype score statistics [40]). However, these tests are powerful when selection acts on a new rather than an existing mutation [30], [31] and when selection acts on a dominant or codominant allele [41]. In the case of SNP rs11868112, the beneficial allele in cold climates segregates at appreciable frequencies in sub-Saharan African populations (8%–43%), thus suggesting that this variant predates the dispersal of human populations out of Africa and that this allele was neutral before becoming advantageous when humans moved to colder climates. Modeling studies have shown that under these circumstances standard neutrality tests have inadequate power to detect a signal of selection. For example, simulations of a model of directional selection on standing variation determined that, if a neutral allele occurred at frequency greater than 5% prior to becoming advantageous, virtually no signature is expected on the frequency spectrum, on patterns of linkage disequilibrium and on polymorphism levels [31]. Given the relatively high frequency of the derived allele at rs11868112 in sub-Saharan African populations, it is therefore not surprising that we detected a signature of natural selection only by using the climate correlation approach.

The molecular function of *RPTOR* is consistent with a role in local adaptations. This is because the MTORC1 complex, which contains RPTOR as a critical component, integrates environmental signals to regulate cell growth, metabolism and survival ([42] and references therein). However, given the diversity of biological processes regulated by MTORC1 and by retinoids, it is difficult to pinpoint the function of RPTOR that is the most likely target of selection. One possibility is that SNP rs11868112 influences the regulation of energy metabolism and mitochondrial function; under this model, the derived allele would have conferred a selective advantage by increasing thermogenesis during the dispersal to progressively colder climates. This scenario is supported by the fact that the MTOR pathway controls mitochondrial function, especially ATP synthetic capacity [43], directly [44] and indirectly [45]. Tissue-specific knockouts (KOs) of *raptor* have provided further support for the critical role of mTORC1 signaling on whole body metabolism. Adipose-specific *raptor* KO mice are resistant to diet-induced obesity, due to an increased mitochondrial uncoupling in white adipose tissue [46], [47]. Because mitochondrial uncoupling is an important mechanism for generating body heat, it is possible that the selective advantage conferred by SNP rs11868112 is due to its effect on thermogenesis and cold tolerance. Adaptations to cold climates are evident in the geographic distributions of many traits. For example, significant correlations exist between body mass and temperature [10], [48], consistent with the long standing hypotheses that variation in body size and proportions are adaptations to maintain temperature homeostasis [49], [50]. Furthermore, there is evidence that human metabolism has been shaped by adaptations to cold stress from studies of arctic populations, which exhibit elevated basal metabolic rates compared to non-indigenous populations [11].

Although these geographic patterns of human phenotypes are well established, the genetic factors contributing to these adaptations are only partially understood. Genetic variants that can increase mitochondrial uncoupling efficiency might be advantageous in cold climates and thus may have been selected during human migrations northward. The derived T allele rs11868112, which is associated with lower RA-dependent transcription levels, may result in increased mitochondrial uncoupling in adipose tissue and, hence, higher thermogenesis. Consistent with the hypothesis that this allele confers resistance to cold stress, it increases in frequency with decreasing winter temperatures in worldwide population samples, and it is relatively rare in the equatorial populations of sub-Saharan Africa and South East Asia (see Figure 2). Although a role for RA in the transcriptional induction of *RPTOR* was not previously reported, retinoids have been long known to regulate thermogenesis and energy expenditure through their effects on the expression of the major uncoupling protein gene, *UCP1*, in brown adipose tissue (BAT) [51]. This protein plays a key role in nonshivering thermogenesis, which is the main mechanism for heat generation in human infants. As with the *RPTOR* gene, a distal enhancer upstream to the proximal *UCP1* promoter was found to contain a RAR response element, which mediates its transcriptional induction [36], [52]. Therefore, our finding that the transcription of the *RPTOR* gene is induced by RA treatment in liver and breast epithelial cells is consistent with the known function of RAR in regulating the expression of another gene with a clear role in thermogenesis. More recently, experiments in mice identified a role for retinoids in adipose tissue remodeling and, more specifically, in the acquisition of BAT-like properties in white adipose tissue [53]. These findings further support the notion that the biological functions of RAR include the regulation of energy balance and thermogenesis. Moreover, our observation that *RPTOR* is induced by retinoids provides a possible mechanistic link connecting the action of retinoids in adipose tissue remodeling and the finding that the adipose-specific *raptor* KO exhibits increased mitochondrial uncoupling (i.e. a typical BAT property) in white adipose tissue.

Another possible explanation for the signal of selection observed at SNP rs11868112 may be related to the role of the MTOR pathway in the regulation of the immune response. Indeed, the MTOR pathway plays multiple roles in immunity, especially in the activation and proliferation of T cells [20], and has been implicated in the etiology of autoimmune disorders, such as systemic lupus erythematosus (SLE) [54]. Moreover, the specific inhibitors for this pathway, rapamycin and its derivatives, can decrease proliferation of T lymphocytes and are used as immunosuppressant to avoid allograft rejection [20] or to treat autoimmune patients [55], [56]. Since pathogen diversity decreases with latitude mainly as a result of climatic factors [57], the optimal level of immune response is also expected to vary according to latitude and climate. Therefore, we hypothesize that the increase in the frequency of the derived T allele with decreasing temperatures is due to selective pressures acting on the MTORC1 function in the regulation of the immune response. Under this scenario, the decrease in *RPTOR* expression associated with the T allele at high latitudes could reflect a shift to maintain the appropriate balance between pathogen pressures and immune response, with an exaggerated immune response possibly resulting in increased risk for autoimmune diseases.

It might be argued that the modest (∼20%) decrease of *RPTOR* expression associated with the T allele is not sufficient to generate significant phenotypic and fitness differences between arctic and tropical populations. One possibility is that RPTOR and the MTOR pathway are located at the top of the signaling cascade [19], therefore, a subtle change in its expression can have major consequences. Alternatively, as observed for most susceptibility SNPs identified through GWAS of common diseases, rs11868112 may be just one of many SNPs with small effects on the phenotypes that are adaptive in different climates.

## Materials and Methods

### Data mining

Genotype data in the *RPTOR* gene for HGDP individuals was obtained from published Illumina HumanMap 650Y data (http://hagsc.org/hgdp/files.html). The genotype data for the same SNPs from four HapMap Phase III populations (Luhya, Maasai, Tuscans, and Gujarati) (http://www.hapmap.org) and five additional populations (Vasekela !Kung from South Africa, lowland Amhara from Ethiopia, Naukan Yup'ik and Maritime Chukchee from Siberia, and Australian Aborigines) were also incorporated (Hancock and Di Rienzo, personal communication). In total, 61 human populations were included in the current study. This study uses the Bayesian geographic analyses method of Coop et al. (2010) [27], which is a model-based method that tests whether a linear relationship between allele frequency and a variable provides a significantly better fit to the data than the null model alone (where the null model is given by a matrix of the covariance of allele frequencies between populations). The environmental variables included latitude and seven climate variables in the summer and winter seasons.

### Re-sequencing and analysis

Twenty-four unrelated Hapmap samples (8 YRI, 8 CEU and 8 ASN) were chosen for re-sequencing the coding regions and 48 unrelated HapMap samples (16 YRI, 16 CEU, and 16 ASN) for re-sequenced in the non-coding regions. cDNA was synthesized from RNA extracted from the lymphoblastoid cell lines of the HapMap samples using the Super Transcript III First-Strand Synthesis System for RT-PCR (Invitrogen, Carlsbad, CA) and utilized as template. Conserved non-coding regions were identified by using the ECR genome browser (http://ecrbrowser.dcode.org/) and by choosing regions conserved between human and at least two additional species (see Figure S3). A 2.6 kb segment spanning SNP rs11868112 as well as the *RPTOR* promoter were also included in the resequencing survey. PCR was performed by using the primers in Table S5. After exonuclease I and Shrimp Alkaline Phosphatase (United States Biochemicals, Cleveland, OH) treatment, sequencing was performed by using internal primers in Table S5 and BigDye Terminator v3.1 (Applied Biosystems, Foster City, CA). In total, 34.4 kb (6.0 kb for coding and 28.4 kb for non-coding) were amplified and re-sequenced. Polymorphisms were scored by PolyPhred [58] and confirmed visually. Visual genotype and LD between SNPs were determined by using the Genome Variation Server (http://gvs.gs.washington.edu/GVS/). Population genetics indices, including segregating sites (S), nucleotide diversity (π) [59], Watterson's estimator of the population mutation rate parameter (θ_w_) [60], Tajima's D [61], were calculated by Slider (http://genapps.uchicago.edu/labweb/index.html). The expected distribution of nucleotide diversity and Tajima's D was generated by coalescent simulations using the software ms [62] with appropriate demographic models [63]. All re-sequencing data will be made publicly available in PharmGKB (http://www.pharmgkb.org).

### Cell culture

The human hepatocellular carcinoma cell line HepG2 was cultured in minimum essential medium (MEM, ATCC, Manassas, VA) supplemented with 10% fetal bovine serum (FBS, Invitrogen, Carlsbad, CA). The human breast cancer cell line MCF-7 was maintained in Dulbecco's Modified Eagle's Medium (DMEM; Invitrogen) with 10% FBS and 0.1% insulin (Sigma, St. Louis, MO). Before any AM580 or DMSO treatment, cells were grown for 48 hrs in medium with 10% charcoal-stripped FBS (Invitrogen).

### Quantification of *RPTOR* mRNA expression

Cells were treated with 100 nM AM580 (Sigma) or DMSO (Sigma) for 2hrs, 4hrs, 8hrs, 12hrs, 24hrs, and 48hrs, and then harvested. RNA was extracted using the RNeasy Mini Kit (Qiagen, Valencia, CA) and cDNAs were synthesized with the High Capacity Reverse Transcription Kit (Applied Biosystems). *RPTOR* mRNA levels were determined by real time PCR using the *power* SYBR green (Applied Biosystems) with primers 5′-CGGGGAGGTCTGGGTCTTCAA-3′ and 5′-CTCCTGCTCCCGCTGTAGTGC-3′ [64]. *β-actin* was used as a calibrator gene in real time PCR with the primers 5′-ACGTGGACATCCGCAAAGAC-3′ and 5′-CAAGAAAGGGTGTAACGCAACTA-3′ [65]. For each of three independent biological replicates, three technical replicates were performed for each time point on a StepOne Plus Realtime PCR System (Applied Biosystems).

### ChIP-PCR

ChIP was carried out using the ChIP Assay Kit (Upstate, Indianapolis, IN) according to the manufacturer's protocol. Briefly, 10^7^ cells grown for 48 hours in medium with charcoal-stripped FBS and then treated with 100nM AM580 or DMSO for 1 hr, were incubated for 10 minutes with 1% formaldehyde at room temperature. The fixed cells were treated with 1.25 M glycine for 5 minutes, washed twice with ice-cold phosphate buffered saline (Invitrogen) containing protease inhibitor cocktail (PIC, Sigma) and phenylmethylsulfonyl fluoride (PMSF, Fisher, Pittsburgh, PA), scraped, lysed and sonicated to obtain 200–800 bp fragments with the Sonicator 4000 (MISONIX, Farmingdale, NY). The solubilized chromatin was diluted 10-fold with dilution buffer, and pre-cleared with protein A beads. After centrifuging and transferring the supernatant, 1% sample was stored as input and the remaining chromatin was incubated with rabbit polyclonal anti-POU2F1 (sc-232X) or anti-RARA (sc-551X; Santa Cruz Biotechnology, Santa Cruz, CA) and normal rabbit IgG (Santa Cruz Biotechnology) and immunoprecipitated with protein A beads. After washing with low salt, high salt, LiCl and TE buffer twice, the immunoprecipitated chromatin was eluted and de-crosslinked. Upon proteinase K treatment (Qiagen) DNA was recovered by QIAquick PCR purification kit (Qiagen). The obtained DNA was quantified by real time PCR with iQ SYBR green (Bio-Rad, Hercules, CA) and primer pairs 5′- AGGTCTGCAACACAGCACAT -3′ and 5′- CTGGGAGCTATGCCTGGTC -3′, and 5′-CTAAGTGCTGGGTCGTAAGTTGT-3′ and 5′-GAATGCAGGCTATAAATCAGGAG-3′ to quantify the enrichment for POU2F1 and RARA binding site, respectively. For each ChIP assay, three technical replicates were performed for three biological replicates on a StepOne Plus Realtime PCR System (Applied Biosystems).

### Luciferase reporter gene assays

A 3.7 kb segment containing the derived T allele of rs11868112 and the putative RAR binding site (see Figure S4) was amplified by nested PCR. In the first round of PCR the primers 5′-TTGCGAAAGTAAATGCTAT-3′ and 5′-CAGAGGGGCCTTGAGATGACCA-3′ were used. In the second round of PCR the primers 5′-CAGTC-GCTAGC-TTCCCTCACTCTGTCCCCCAATG-3′ and 5′-CAGTC-CTCGAG-TTCCTGACCTGCCAAATCTGTG-3′ were used to append the PCR fragment with *Nhe*I and *Xho*I restriction sites, respectively. For both PCR reactions, iProof High-Fidelity DNA Polymerase (Bio-Rad) was used to avoid the introduction of mutations. After digestion with *Nhe*I and *Xho*I (New England Biolabs, Ipswich, MA), the DNA fragment was cloned into the pGL3-promoter vector (Promega, Hercules, CA). The plasmid with the ancestral allele (C) was generated with the QuikChange Site-Directed Mutagenesis Kit (Stratagene, La Jolla, CA) using primer pair 5′-GCCCTTGACAAGCT**C**ACAAACTTGTAGGAGGG-3′ **and 5′-CCCTCCTACAAGTTTGT**G**AGCTTGTCAAGGGC-3′**(target in bold) according to the manufacturer's recommendations. All plasmids were verified by sequencing prior to following experiments.****

Two million HepG2 cells or 2.5*10^6^ MCF-7 cells were seeded into 10-cm plates 24 hours before transfection. Plasmid DNA (9.5 µg) was transfected using FuGene HD (Roche, Indianapolis, IN) according to the manufacturer's recommendations. Plasmid pRL-TK (Promega) DNA (0.5 µg) was co-transfected as internal control. Twenty-four hours after transfection, cells were split, cultured for 12h, and starved for 48 hours in medium with charcoal-stripped FBS. After treatment with 100nM AM580 or DMSO for 2hrs, 4hrs, 8hrs, 12hrs, 24hrs, and 48hrs, cells were harvested and luciferase activity was determined using the Dual-Luciferase Reporter Assay System (Promega) according to the manufacturer's protocol. The enhancer activity was determined as the ratio between Firefly and *Renilla* luciferase activity. Four independent replicates were performed for each experiment.

### Statistical analyses

For all analyses of expression, POU2F1 and RARA binding, and reporter gene data, we used independent two-tailed *t*-tests in SPSS 15.0 (SPSS Inc., Chicago, IL) and the null hypothesis was rejected when *P*<0.05.

## Supporting Information

Figure S1 Visual genotype for *RPTOR* coding region resequencing. Each column indicates one SNP while each array denotes one individual. Blue, red, and yellow represent homozygous of common allele, heterozygous, and homozygous of rare allele, respectively. Af, Eu, and As indicate YRI, CEU, and ASN HapMap populations, respectively. All positions refer to *RPTOR* mRNA sequence (Genbank ID NM_020761).
(0.05 MB TIF)

Figure S2 Visual genotype for *RPTOR* non-coding region resequencing. Each column indicates one SNP while each array denotes one individual. Blue, red, yellow, and grey represent homozygous of common allele, heterozygous, homozygous of rare allele, and missing data, respectively. Af, Eu, and As indicate YRI, CEU, and ASN HapMap populations, respectively. All positions refer to the genome sequence (build 36) for chromosome 17.
(2.18 MB TIF)

Figure S3 Seleted region for *RPTOR* non-coding resequencing in this study. The human *RPTOR* gene cluster is aligned to frog, fish, chicken, opossum, mouse, dog, and macaque (from top to bottom) genomes by ECR browser (http://ecrbrowser.dcode.org/). The vertical bar on top represents surveyed regions while red, blue, and green indicate conserved non-coding region, promoter, and rs11868112 nearby region, respectively. The sequence identity ranges from 50% to 100% and is displayed by the height of the peak while green, blue, salmon, red, and yellow denote repeat, coding, intronic, intergenic, and untranslated region, respectively.
(0.13 MB TIF)

Figure S4 Schematic figure for the enhancer ∼25kb upstream of *RPTOR*. The black bars from left to right indicate POU2F1, RAR binding sites and *RPTOR* gene, respectively. The distances between them are displayed below. The primer position for ChIP-real time PCR, the SNP in predicted POU2F1 binding site is also provided.
(0.03 MB TIF)

Table S1 Derived allele frequencies of the *RPTOR* SNPs in the different human populations.
(0.15 MB XLS)

Table S2 Bayes Factors for the *RPTOR* SNPs in the HGDP panel.
(0.55 MB DOC)

Table S3 Bayes Factors empirical p values for the *RPTOR* SNPs in the HGDP panel.
(0.55 MB DOC)

Table S4 Population index for the resequenced regions in YRI (A), CEU (B), and the ASN (C) populations.
(0.08 MB DOC)

Table S5 Primers for *RPTOR* PCR and resequencing in this study.
(0.05 MB DOC)
