# Supplementary figures and images for: Allele-Specific Down-Regulation of RPTOR Expression Induced by Retinoids Contributes to Climate Adaptations
Source: PLoS Genet. 2010 Oct 28;6(10):e1001178. doi: 10.1371/journal.pgen.1001178 (PMC2965758; doi:10.1371/journal.pgen.1001178)

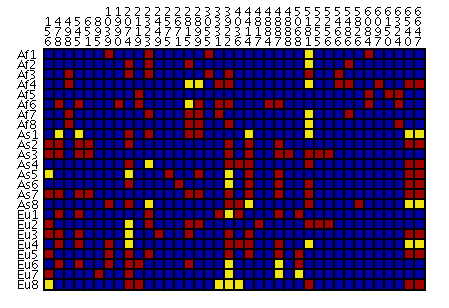

Supplement: Figure S1 — Visual genotype for RPTOR coding region resequencing. Each column indicates one SNP while each array denotes one individual. Blue, red, and yellow represent homozygous of common allele, heterozygous, and homozygous of rare allele, respectively. Af, Eu, and As indicate YRI, CEU, and ASN HapMap populations, respectively. All positions refer to RPTOR mRNA sequence (Genbank ID NM_020761). (0.05 MB TIF) [file pgen.1001178.s001.tif]

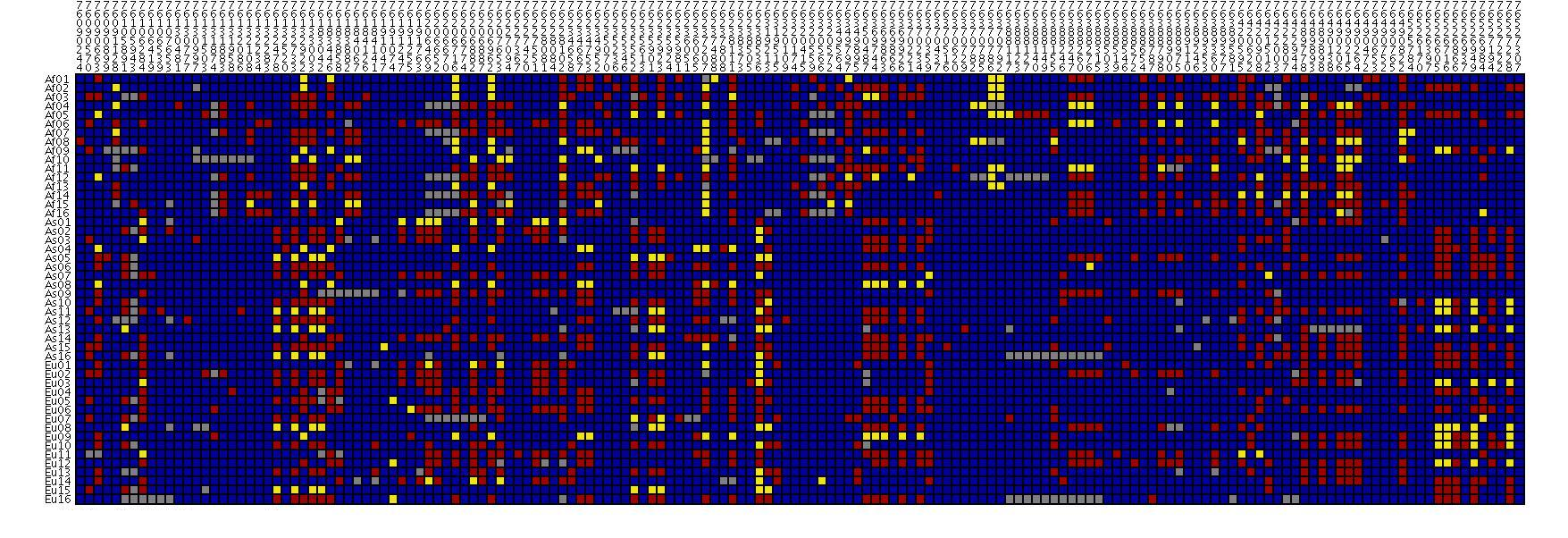

Supplement: Figure S2 — Visual genotype for RPTOR non-coding region resequencing. Each column indicates one SNP while each array denotes one individual. Blue, red, yellow, and grey represent homozygous of common allele, heterozygous, homozygous of rare allele, and missing data, respectively. Af, Eu, and As indicate YRI, CEU, and ASN HapMap populations, respectively. All positions refer to the genome sequence (build 36) for chromosome 17. (2.18 MB TIF) [file pgen.1001178.s002.tif]

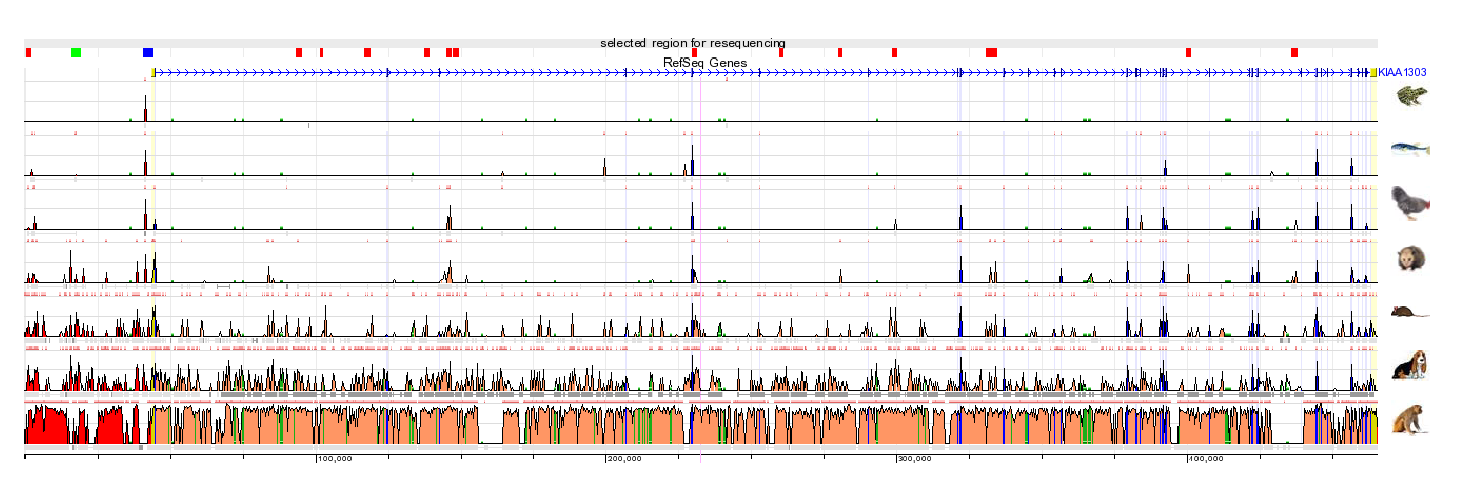

Supplement: Figure S3 — Seleted region for RPTOR non-coding resequencing in this study. The human RPTOR gene cluster is aligned to frog, fish, chicken, opossum, mouse, dog, and macaque (from top to bottom) genomes by ECR browser (http://ecrbrowser.dcode.org/). The vertical bar on top represents surveyed regions while red, blue, and green indicate conserved non-coding region, promoter, and rs11868112 nearby region, respectively. The sequence identity ranges from 50% to 100% and is displayed by the height of the peak while green, blue, salmon, red, and yellow denote repeat, coding, intronic, intergenic, and untranslated region, respectively. (0.13 MB TIF) [file pgen.1001178.s003.tif]

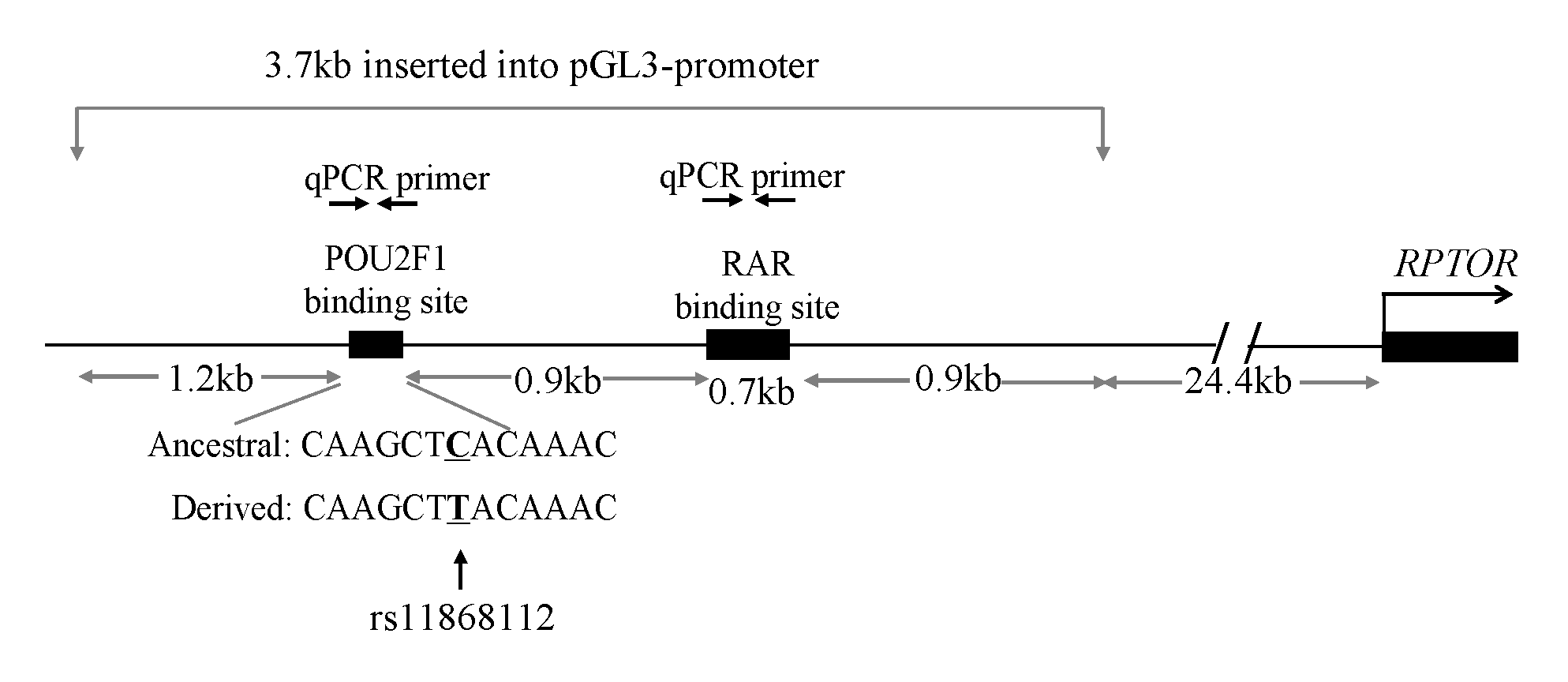

Supplement: Figure S4 — Schematic figure for the enhancer ∼25kb upstream of RPTOR. The black bars from left to right indicate POU2F1, RAR binding sites and RPTOR gene, respectively. The distances between them are displayed below. The primer position for ChIP-real time PCR, the SNP in predicted POU2F1 binding site is also provided. (0.03 MB TIF) [file pgen.1001178.s004.tif]
