# Supplementary material for: Allele-Specific Down-Regulation of RPTOR Expression Induced by Retinoids Contributes to Climate Adaptations
Source: PLoS Genet. 2010 Oct 28;6(10):e1001178. doi: 10.1371/journal.pgen.1001178 (PMC2965758; doi:10.1371/journal.pgen.1001178)
Supplement: Table S2 — Bayes Factors for the RPTOR SNPs in the HGDP panel. (0.55 MB DOC) [file pgen.1001178.s006.doc]

Table S2: Bayes Factors for the *RPTOR* SNPs in the HGDP panel (rs11868112 in bold)

|  | Latitude | Minimum T (summer) | Minimum T (winter) | Maximum T (summer) | Maximum T (winter) | Mean T (summer) | Mean T (winter) | Precipitation rate (summer) | Precipitation rate (winter) | Short-Wave radiation (summer) | Short-Wave radiation (winter) | Relative humidity (summer) | Relative humidity (winter) | PC1 (summer) | PC2 (summer) | PC1 (winter) | PC2 (winter) |
| --- | --- | --- | --- | --- | --- | --- | --- | --- | --- | --- | --- | --- | --- | --- | --- | --- | --- |
| rs4074302 | 2.3E-01 | 3.4E-01 | 1.9E-01 | 3.7E-01 | 4.1E-01 | 3.3E-01 | 2.3E-01 | 1.7E-01 | 1.9E-01 | 1.5E-01 | 2.9E-01 | 1.3E-01 | 5.3E-01 | 1.8E-01 | 1.3E-01 | 1.8E-01 | 2.7E-01 |
| rs8071962 | 1.0E+00 | 5.1E+00 | 4.3E+00 | 2.1E+00 | 8.2E+00 | 3.7E+00 | 5.4E+00 | 8.7E-01 | 4.7E-01 | 1.7E-01 | 1.8E+00 | 5.9E-01 | 3.4E-01 | 7.0E-01 | 7.1E-01 | 2.8E+00 | 4.1E-01 |
| rs4890025 | 2.7E+00 | 1.7E-01 | 8.7E-01 | 4.0E-01 | 1.8E+00 | 2.8E-01 | 1.1E+00 | 2.6E-01 | 1.6E-01 | 1.2E+00 | 5.7E+00 | 3.5E-01 | 1.9E-01 | 2.8E-01 | 1.2E-01 | 9.8E-01 | 1.2E-01 |
| rs7503219 | 4.1E+00 | 1.4E-01 | 4.1E-01 | 1.5E-01 | 3.9E-01 | 1.4E-01 | 3.4E-01 | 1.9E-01 | 2.4E+00 | 2.2E-01 | 8.8E-01 | 1.4E-01 | 1.5E-01 | 9.3E-02 | 1.0E-01 | 3.0E-01 | 2.7E-01 |
| rs10931 | 2.8E-01 | 1.5E-01 | 2.4E-01 | 4.1E-01 | 6.8E-01 | 2.7E-01 | 3.3E-01 | 2.2E-01 | 1.6E+00 | 2.0E-01 | 8.7E-01 | 1.9E-01 | 2.1E-01 | 2.0E-01 | 8.9E-02 | 2.2E-01 | 4.5E-01 |
| rs12943620 | 2.2E-01 | 1.6E-01 | 2.2E-01 | 2.4E-01 | 2.5E-01 | 2.5E-01 | 2.2E-01 | 7.0E-01 | 2.8E-01 | 1.7E-01 | 2.0E-01 | 7.7E-01 | 1.4E-01 | 2.3E-01 | 1.1E-01 | 1.2E-01 | 1.5E-01 |
| rs11869626 | 6.2E-01 | 1.3E-01 | 3.6E-01 | 1.4E-01 | 3.6E-01 | 1.4E-01 | 3.0E-01 | 7.7E-01 | 3.3E-01 | 2.1E-01 | 4.1E-01 | 1.6E-01 | 1.5E-01 | 9.4E-02 | 9.5E-02 | 2.0E-01 | 1.6E-01 |
| rs11657796 | 2.2E-01 | 1.1E-01 | 1.7E-01 | 1.2E-01 | 1.7E-01 | 1.1E-01 | 1.6E-01 | 1.8E-01 | 2.0E-01 | 2.1E-01 | 2.2E-01 | 1.3E-01 | 1.2E-01 | 7.0E-02 | 8.3E-02 | 9.1E-02 | 1.2E-01 |
| rs7213201 | 9.5E-01 | 1.5E-01 | 3.8E-01 | 1.6E-01 | 3.4E-01 | 1.5E-01 | 3.2E-01 | 3.9E-01 | 4.1E-01 | 2.7E-01 | 5.1E-01 | 2.2E-01 | 1.4E-01 | 1.0E-01 | 1.3E-01 | 2.7E-01 | 1.5E-01 |
| rs4453556 | 3.6E-01 | 1.3E-01 | 2.7E-01 | 1.8E-01 | 2.0E-01 | 1.7E-01 | 2.1E-01 | 2.0E-01 | 2.1E-01 | 2.2E-01 | 2.3E-01 | 2.0E-01 | 2.8E-01 | 1.3E-01 | 9.8E-02 | 1.1E-01 | 1.9E-01 |
| rs4561525 | 2.0E+00 | 1.4E-01 | 4.9E-01 | 1.4E-01 | 1.8E+00 | 1.4E-01 | 9.0E-01 | 6.4E-01 | 1.7E-01 | 1.5E-01 | 3.7E+00 | 2.7E-01 | 1.4E-01 | 9.7E-02 | 1.3E-01 | 1.0E+00 | 1.0E-01 |
| rs11650988 | 1.9E-01 | 1.3E-01 | 3.3E-01 | 1.3E-01 | 3.2E-01 | 1.2E-01 | 3.2E-01 | 2.1E-01 | 2.3E-01 | 2.1E-01 | 1.7E-01 | 1.9E-01 | 1.3E-01 | 8.4E-02 | 1.3E-01 | 1.6E-01 | 1.4E-01 |
| rs7212223 | 8.8E+00 | 1.7E-01 | 1.4E+00 | 1.4E-01 | 2.2E+00 | 1.6E-01 | 1.7E+00 | 3.0E+00 | 5.0E-01 | 2.6E-01 | 3.5E+01 | 1.8E-01 | 1.7E-01 | 9.2E-02 | 3.2E-01 | 2.4E+00 | 1.2E-01 |
| rs7216808 | 4.9E-01 | 1.3E-01 | 2.8E-01 | 1.6E-01 | 2.4E-01 | 1.5E-01 | 2.5E-01 | 2.1E-01 | 3.1E-01 | 1.8E-01 | 3.4E-01 | 1.3E-01 | 1.3E-01 | 9.8E-02 | 9.8E-02 | 1.7E-01 | 1.2E-01 |
| rs12601738 | 3.1E+02 | 1.4E-01 | 7.5E+00 | 1.4E-01 | 8.2E+01 | 1.3E-01 | 1.4E+01 | 6.2E-01 | 6.8E-01 | 2.1E-01 | 6.2E+01 | 8.7E-01 | 1.2E-01 | 1.2E-01 | 1.6E-01 | 1.9E+02 | 2.0E-01 |
| **rs11868112** | 3.6E+02 | 1.5E-01 | 2.4E+05 | 5.7E-01 | 1.5E+06 | 1.9E-01 | 6.7E+05 | 2.4E+00 | 3.5E+00 | 2.3E-01 | 2.2E+01 | 4.8E-01 | 1.8E-01 | 1.1E+00 | 1.9E-01 | 3.5E+06 | 3.5E-01 |
| rs7224003 | 2.3E-01 | 1.1E-01 | 3.3E-01 | 1.3E-01 | 3.4E-01 | 1.2E-01 | 3.2E-01 | 1.7E-01 | 2.1E-01 | 2.0E-01 | 1.9E-01 | 2.2E-01 | 1.3E-01 | 8.6E-02 | 9.9E-02 | 1.5E-01 | 1.3E-01 |
| rs4073061 | 3.2E-01 | 6.8E-01 | 3.7E-01 | 9.0E-01 | 5.7E-01 | 7.6E-01 | 4.5E-01 | 1.8E-01 | 2.2E-01 | 3.4E-01 | 3.3E-01 | 1.5E-01 | 2.2E-01 | 3.8E-01 | 1.7E-01 | 2.7E-01 | 1.8E-01 |
| rs4890037 | 5.5E-01 | 1.3E-01 | 3.1E-01 | 1.3E-01 | 4.5E-01 | 1.2E-01 | 3.8E-01 | 1.2E+00 | 9.4E-01 | 1.5E-01 | 7.1E-01 | 1.4E+00 | 1.5E-01 | 8.8E-02 | 1.7E-01 | 2.6E-01 | 2.2E-01 |
| rs4889856 | 1.0E+01 | 1.6E-01 | 8.8E+01 | 1.7E-01 | 2.3E+01 | 1.7E-01 | 6.8E+01 | 4.4E-01 | 1.9E+00 | 1.7E-01 | 1.4E+00 | 5.6E-01 | 5.4E-01 | 1.2E-01 | 9.8E-02 | 1.5E+01 | 2.5E+00 |
| rs3923514 | 2.2E-01 | 1.7E-01 | 1.8E-01 | 1.8E-01 | 2.1E-01 | 1.8E-01 | 1.8E-01 | 1.7E-01 | 1.5E-01 | 1.5E-01 | 3.2E-01 | 1.4E-01 | 3.6E-01 | 1.0E-01 | 1.0E-01 | 1.2E-01 | 1.4E-01 |
| rs8075710 | 2.6E-01 | 4.0E-01 | 6.1E-01 | 4.8E-01 | 4.4E-01 | 4.1E-01 | 4.9E-01 | 2.6E-01 | 1.5E-01 | 5.9E-01 | 2.9E-01 | 1.5E-01 | 1.4E-01 | 1.5E-01 | 1.5E-01 | 2.2E-01 | 1.1E-01 |
| rs4627412 | 3.9E-01 | 1.5E-01 | 2.3E-01 | 1.4E-01 | 2.7E-01 | 1.5E-01 | 2.3E-01 | 1.8E-01 | 2.2E-01 | 1.9E-01 | 3.7E-01 | 1.6E-01 | 1.8E-01 | 8.6E-02 | 1.1E-01 | 1.6E-01 | 1.2E-01 |
| rs9911978 | 3.2E-01 | 1.5E-01 | 2.3E-01 | 1.5E-01 | 4.3E-01 | 1.6E-01 | 2.4E-01 | 2.4E-01 | 2.2E-01 | 1.8E-01 | 2.8E-01 | 1.6E-01 | 1.9E-01 | 9.4E-02 | 1.1E-01 | 2.0E-01 | 1.2E-01 |
| rs4890042 | 3.3E-01 | 1.0E-01 | 2.2E-01 | 1.2E-01 | 3.7E-01 | 1.1E-01 | 2.3E-01 | 1.5E-01 | 3.0E-01 | 2.6E-01 | 5.2E-01 | 2.0E-01 | 3.4E-01 | 8.6E-02 | 8.7E-02 | 2.2E-01 | 2.6E-01 |
| rs12937147 | 7.4E-01 | 1.3E-01 | 1.7E+00 | 1.3E-01 | 9.9E+00 | 1.3E-01 | 3.1E+00 | 2.1E-01 | 1.5E-01 | 1.3E-01 | 2.3E+00 | 2.4E-01 | 1.9E-01 | 8.9E-02 | 9.6E-02 | 4.1E+00 | 1.0E-01 |
| rs7209040 | 3.7E-01 | 1.3E-01 | 8.5E-01 | 1.5E-01 | 1.6E+00 | 1.4E-01 | 2.1E+00 | 5.3E-01 | 1.6E-01 | 1.7E-01 | 4.3E-01 | 3.8E-01 | 1.6E-01 | 1.0E-01 | 1.6E-01 | 3.8E-01 | 1.4E-01 |
| rs11871623 | 3.0E-01 | 1.9E-01 | 4.4E-01 | 2.2E-01 | 4.1E-01 | 2.1E-01 | 4.8E-01 | 2.0E-01 | 1.9E-01 | 1.9E-01 | 2.8E-01 | 2.2E-01 | 1.6E-01 | 1.3E-01 | 1.1E-01 | 2.1E-01 | 1.4E-01 |
| rs7226296 | 3.6E-01 | 1.3E-01 | 7.0E+01 | 1.3E-01 | 1.0E+03 | 1.3E-01 | 4.2E+02 | 2.1E-01 | 1.5E-01 | 1.8E-01 | 6.1E-01 | 2.3E-01 | 1.4E-01 | 8.1E-02 | 1.0E-01 | 1.0E+02 | 1.1E-01 |
| rs7215496 | 1.7E+00 | 1.2E-01 | 1.1E+01 | 1.9E-01 | 2.3E+01 | 1.7E-01 | 2.5E+01 | 2.3E-01 | 3.3E-01 | 1.7E-01 | 8.7E-01 | 4.3E-01 | 1.3E-01 | 1.5E-01 | 1.1E-01 | 9.4E+00 | 1.5E-01 |
| rs4889863 | 3.0E-01 | 1.5E-01 | 2.1E-01 | 1.5E-01 | 2.4E-01 | 1.4E-01 | 2.1E-01 | 2.8E-01 | 1.5E-01 | 2.3E-01 | 6.3E-01 | 1.7E-01 | 2.3E-01 | 8.4E-02 | 1.5E-01 | 1.3E-01 | 1.2E-01 |
| rs12951309 | 2.1E+00 | 1.3E-01 | 1.2E+00 | 1.4E-01 | 3.8E+00 | 1.3E-01 | 1.8E+00 | 2.2E-01 | 1.6E-01 | 1.8E-01 | 4.1E+00 | 3.3E-01 | 2.0E-01 | 1.1E-01 | 1.2E-01 | 2.1E+00 | 1.1E-01 |
| rs12949279 | 2.0E+00 | 1.3E-01 | 6.4E-01 | 1.6E-01 | 2.1E+00 | 1.5E-01 | 9.8E-01 | 1.8E-01 | 1.7E-01 | 1.7E-01 | 6.3E+00 | 1.6E-01 | 6.2E-01 | 9.5E-02 | 9.5E-02 | 1.3E+00 | 1.4E-01 |
| rs7210046 | 4.2E-01 | 4.6E-01 | 3.5E-01 | 3.9E-01 | 3.9E-01 | 4.0E-01 | 3.7E-01 | 4.3E-01 | 3.1E-01 | 3.5E-01 | 3.5E-01 | 1.6E-01 | 2.2E-01 | 2.0E-01 | 1.4E-01 | 2.0E-01 | 2.4E-01 |
| rs11653499 | 5.3E-01 | 1.3E-01 | 6.2E-01 | 1.3E-01 | 2.3E+00 | 1.2E-01 | 1.3E+00 | 1.8E-01 | 1.5E-01 | 2.8E-01 | 6.6E-01 | 1.5E-01 | 3.0E-01 | 8.1E-02 | 1.0E-01 | 9.3E-01 | 1.6E-01 |
| rs4890055 | 2.2E-01 | 1.8E-01 | 1.5E-01 | 1.3E-01 | 1.8E-01 | 1.2E-01 | 1.5E-01 | 1.8E-01 | 1.7E-01 | 4.4E-01 | 7.2E-01 | 6.6E-01 | 2.2E-01 | 9.2E-02 | 1.8E-01 | 1.0E-01 | 1.2E-01 |
| rs9890502 | 1.0E+00 | 1.6E-01 | 6.1E-01 | 2.2E-01 | 2.3E+00 | 1.7E-01 | 1.2E+00 | 1.8E-01 | 1.6E-01 | 1.8E-01 | 1.4E+00 | 1.3E-01 | 1.9E-01 | 1.1E-01 | 1.0E-01 | 1.1E+00 | 1.2E-01 |
| rs7503807 | 7.9E+00 | 1.3E-01 | 2.2E+00 | 1.4E-01 | 7.1E+00 | 1.3E-01 | 4.0E+00 | 2.3E-01 | 3.7E-01 | 2.0E-01 | 8.0E+00 | 1.9E-01 | 7.0E-01 | 8.3E-02 | 9.9E-02 | 9.4E+00 | 1.4E-01 |
| rs9903842 | 6.0E-01 | 2.7E-01 | 3.0E+00 | 2.1E-01 | 1.8E+00 | 2.2E-01 | 3.0E+00 | 2.7E-01 | 2.6E-01 | 2.6E-01 | 4.3E-01 | 1.4E-01 | 1.7E-01 | 1.2E-01 | 2.9E-01 | 7.2E-01 | 1.7E-01 |
| rs901065 | 3.8E+00 | 1.7E-01 | 3.1E-01 | 1.6E-01 | 6.6E-01 | 2.7E-01 | 3.7E-01 | 3.1E-01 | 7.5E-01 | 2.1E-01 | 1.3E+01 | 2.0E-01 | 1.5E-01 | 2.0E-01 | 9.0E-02 | 4.6E-01 | 1.5E-01 |
| rs8071015 | 2.1E+00 | 1.6E-01 | 1.4E+00 | 2.2E-01 | 2.6E+00 | 1.9E-01 | 1.7E+00 | 3.4E-01 | 1.5E-01 | 2.1E-01 | 8.2E+00 | 1.6E-01 | 2.6E-01 | 1.1E-01 | 1.0E-01 | 1.2E+00 | 1.3E-01 |
| rs999977 | 1.9E-01 | 1.8E-01 | 1.8E-01 | 2.2E-01 | 1.6E-01 | 2.4E-01 | 1.8E-01 | 1.6E-01 | 1.7E-01 | 1.4E-01 | 1.6E-01 | 1.1E-01 | 1.5E-01 | 1.1E-01 | 1.2E-01 | 9.1E-02 | 1.2E-01 |
| rs12940622 | 1.4E+01 | 1.8E-01 | 1.6E+00 | 2.5E-01 | 4.5E+00 | 2.2E-01 | 3.3E+00 | 1.9E-01 | 2.5E-01 | 1.9E-01 | 5.2E+01 | 1.4E-01 | 2.7E-01 | 1.4E-01 | 1.2E-01 | 5.9E+00 | 1.1E-01 |
| rs9900506 | 5.6E-01 | 1.7E-01 | 2.9E-01 | 1.6E-01 | 2.9E-01 | 1.6E-01 | 2.7E-01 | 1.8E-01 | 3.0E-01 | 1.7E-01 | 2.2E-01 | 3.6E-01 | 2.5E-01 | 9.9E-02 | 1.1E-01 | 1.5E-01 | 1.8E-01 |
| rs7212142 | 1.4E+01 | 1.7E-01 | 4.9E+01 | 1.3E-01 | 3.9E+00 | 1.3E-01 | 3.7E+01 | 2.3E-01 | 2.7E-01 | 2.7E-01 | 7.1E-01 | 1.0E+00 | 1.5E-01 | 8.4E-02 | 1.7E-01 | 2.6E+00 | 1.7E-01 |
| rs12939076 | 2.6E+01 | 1.4E-01 | 1.8E+00 | 1.5E-01 | 2.4E+00 | 1.5E-01 | 1.6E+00 | 2.9E-01 | 7.9E+00 | 1.8E-01 | 1.2E+01 | 9.9E-01 | 1.8E-01 | 1.4E-01 | 1.3E-01 | 1.6E+00 | 4.3E-01 |
| rs9906493 | 2.5E-01 | 4.2E-01 | 3.2E-01 | 1.7E+00 | 6.6E-01 | 6.6E-01 | 4.6E-01 | 2.8E-01 | 1.8E+00 | 3.0E-01 | 6.2E-01 | 1.9E-01 | 3.2E+00 | 8.2E-01 | 1.2E-01 | 2.5E-01 | 5.1E+00 |
| rs4889782 | 3.2E+01 | 1.5E-01 | 4.1E+00 | 1.4E-01 | 5.1E+00 | 1.5E-01 | 7.4E+00 | 1.9E-01 | 1.6E-01 | 3.4E-01 | 1.6E+01 | 1.7E-01 | 1.3E-01 | 8.7E-02 | 9.7E-02 | 3.9E+00 | 1.1E-01 |
| rs9889835 | 5.2E-01 | 4.0E-01 | 3.0E-01 | 4.3E-01 | 4.0E-01 | 4.7E-01 | 3.5E-01 | 2.1E-01 | 1.8E-01 | 2.8E-01 | 3.7E-01 | 1.4E-01 | 1.7E-01 | 1.8E-01 | 1.5E-01 | 2.3E-01 | 1.2E-01 |
| rs1485330 | 3.7E+01 | 1.3E-01 | 3.8E+00 | 1.5E-01 | 8.3E+00 | 1.5E-01 | 3.8E+00 | 2.1E-01 | 1.3E+00 | 1.5E-01 | 5.2E+00 | 4.2E-01 | 1.7E-01 | 1.0E-01 | 1.1E-01 | 4.4E+00 | 3.3E-01 |
| rs6565472 | 7.3E-01 | 1.6E-01 | 9.5E-01 | 1.9E-01 | 1.9E+00 | 1.4E-01 | 1.2E+00 | 1.7E-01 | 1.4E-01 | 2.2E-01 | 2.2E+00 | 2.1E-01 | 2.6E-01 | 1.2E-01 | 1.2E-01 | 7.9E-01 | 1.3E-01 |
| rs7217223 | 7.0E-01 | 1.7E-01 | 4.8E-01 | 1.5E-01 | 3.2E+00 | 1.5E-01 | 9.3E-01 | 2.4E-01 | 1.6E-01 | 2.2E-01 | 4.4E+00 | 1.8E-01 | 3.8E-01 | 1.0E-01 | 1.4E-01 | 1.7E+00 | 1.9E-01 |
| rs4889875 | 3.4E-01 | 1.5E-01 | 2.6E-01 | 1.4E-01 | 2.5E-01 | 1.4E-01 | 2.5E-01 | 1.9E-01 | 6.5E-01 | 1.8E-01 | 2.3E-01 | 2.9E-01 | 1.6E-01 | 9.3E-02 | 1.1E-01 | 1.5E-01 | 2.7E-01 |
| rs9901366 | 2.1E-01 | 4.4E-01 | 2.1E-01 | 5.3E-01 | 1.7E-01 | 5.2E-01 | 2.0E-01 | 2.6E-01 | 2.1E-01 | 1.6E-01 | 1.7E-01 | 1.8E-01 | 2.0E-01 | 3.9E-01 | 1.2E-01 | 1.0E-01 | 1.4E-01 |
| rs7501659 | 2.3E-01 | 1.6E-01 | 1.5E-01 | 1.1E-01 | 1.8E-01 | 1.1E-01 | 1.5E-01 | 1.6E-01 | 4.9E-01 | 2.1E-01 | 2.4E-01 | 3.2E-01 | 1.4E-01 | 7.0E-02 | 1.3E-01 | 9.7E-02 | 1.3E-01 |
| rs9906827 | 1.5E+00 | 1.3E-01 | 1.1E+00 | 2.4E-01 | 5.2E-01 | 1.8E-01 | 6.4E-01 | 3.8E-01 | 2.1E+00 | 2.0E-01 | 3.4E-01 | 1.9E+00 | 7.5E-01 | 2.0E-01 | 1.2E-01 | 3.0E-01 | 7.9E-01 |
| rs9902891 | 3.3E-01 | 1.7E-01 | 3.3E-01 | 1.5E-01 | 3.4E-01 | 1.6E-01 | 3.6E-01 | 1.9E-01 | 1.6E-01 | 1.8E-01 | 2.6E-01 | 2.0E-01 | 1.3E-01 | 8.7E-02 | 1.2E-01 | 1.8E-01 | 1.1E-01 |
| rs7208502 | 2.3E+00 | 1.4E-01 | 1.6E+00 | 3.0E-01 | 6.2E-01 | 2.1E-01 | 8.4E-01 | 3.2E-01 | 1.0E+00 | 2.0E-01 | 4.5E-01 | 3.2E+00 | 4.8E-01 | 3.2E-01 | 1.2E-01 | 4.1E-01 | 7.8E-01 |
| rs12948054 | 8.5E-01 | 1.4E-01 | 1.9E-01 | 1.9E-01 | 2.6E-01 | 2.0E-01 | 2.2E-01 | 1.6E-01 | 1.8E-01 | 1.4E-01 | 6.2E-01 | 1.2E-01 | 1.7E-01 | 1.1E-01 | 8.7E-02 | 1.7E-01 | 1.1E-01 |
| rs4062178 | 2.1E-01 | 1.2E-01 | 1.7E-01 | 3.4E-01 | 1.6E-01 | 2.3E-01 | 1.6E-01 | 2.8E-01 | 1.7E-01 | 1.8E-01 | 1.8E-01 | 1.5E+00 | 3.6E-01 | 3.5E-01 | 9.2E-02 | 8.6E-02 | 2.1E-01 |
| rs1564868 | 1.6E+00 | 1.5E-01 | 9.7E-01 | 1.7E-01 | 5.2E-01 | 1.7E-01 | 6.3E-01 | 2.7E-01 | 9.1E-01 | 1.6E-01 | 5.6E-01 | 3.7E-01 | 2.9E-01 | 1.3E-01 | 1.1E-01 | 4.4E-01 | 3.6E-01 |
| rs7211818 | 2.4E-01 | 1.5E-01 | 1.7E-01 | 2.9E-01 | 2.8E-01 | 1.7E-01 | 1.9E-01 | 1.7E-01 | 2.3E-01 | 1.6E-01 | 4.0E-01 | 2.8E-01 | 4.3E-01 | 1.5E-01 | 9.0E-02 | 1.3E-01 | 2.5E-01 |
| rs6565478 | 1.0E+00 | 1.5E-01 | 1.0E+00 | 1.4E-01 | 7.7E-01 | 1.5E-01 | 8.4E-01 | 1.8E-01 | 3.8E-01 | 1.6E-01 | 5.2E-01 | 2.2E-01 | 1.7E-01 | 9.6E-02 | 1.4E-01 | 5.6E-01 | 1.5E-01 |
| rs12603074 | 3.6E+00 | 1.5E-01 | 9.4E-01 | 1.4E-01 | 2.1E+00 | 1.5E-01 | 1.2E+00 | 2.2E-01 | 2.4E-01 | 1.5E-01 | 5.0E+00 | 4.1E-01 | 1.3E-01 | 9.6E-02 | 9.5E-02 | 1.3E+00 | 1.1E-01 |
| rs9915378 | 8.3E-01 | 1.7E-01 | 8.3E-01 | 1.6E-01 | 2.4E+00 | 1.5E-01 | 1.1E+00 | 4.6E-01 | 1.6E-01 | 1.8E-01 | 1.3E+00 | 4.9E-01 | 1.6E-01 | 1.1E-01 | 1.5E-01 | 1.2E+00 | 1.1E-01 |
| rs9674559 | 2.3E-01 | 1.2E-01 | 1.8E-01 | 2.9E-01 | 2.2E-01 | 1.6E-01 | 1.9E-01 | 4.4E-01 | 2.4E-01 | 2.2E-01 | 3.0E-01 | 1.2E+00 | 5.0E-01 | 2.9E-01 | 1.1E-01 | 1.2E-01 | 3.9E-01 |
| rs4969230 | 1.3E+00 | 1.4E-01 | 1.9E+01 | 1.9E-01 | 4.2E+01 | 1.6E-01 | 1.6E+01 | 2.2E+00 | 5.7E-01 | 2.8E-01 | 6.5E-01 | 8.7E+00 | 2.1E-01 | 2.2E-01 | 2.1E-01 | 9.8E+00 | 2.9E-01 |
| rs8078829 | 4.5E-01 | 2.8E-01 | 4.2E-01 | 2.2E-01 | 3.8E-01 | 2.5E-01 | 3.9E-01 | 1.9E-01 | 1.1E+00 | 2.2E-01 | 2.4E-01 | 1.6E-01 | 1.6E-01 | 1.2E-01 | 1.8E-01 | 1.9E-01 | 3.7E-01 |
| rs7215564 | 2.2E-01 | 1.8E-01 | 1.7E-01 | 1.1E-01 | 1.9E-01 | 1.2E-01 | 1.7E-01 | 1.6E-01 | 8.3E-01 | 1.8E-01 | 1.9E-01 | 3.5E-01 | 1.3E-01 | 6.6E-02 | 1.6E-01 | 9.8E-02 | 1.9E-01 |
| rs9894401 | 3.4E-01 | 1.4E-01 | 2.0E-01 | 1.8E-01 | 2.1E-01 | 1.4E-01 | 1.9E-01 | 5.2E-01 | 1.7E-01 | 8.9E-01 | 1.1E+00 | 4.6E-01 | 5.6E-01 | 1.5E-01 | 2.5E-01 | 1.4E-01 | 2.4E-01 |
| rs7208536 | 2.4E-01 | 1.2E-01 | 1.8E-01 | 1.9E-01 | 1.8E-01 | 1.3E-01 | 1.7E-01 | 3.4E-01 | 1.6E-01 | 5.6E-01 | 3.5E-01 | 6.3E-01 | 4.9E-01 | 1.5E-01 | 1.6E-01 | 1.0E-01 | 1.9E-01 |
| rs11150744 | 1.6E+01 | 1.5E-01 | 3.5E+00 | 2.0E-01 | 1.1E+01 | 1.9E-01 | 4.1E+00 | 2.6E-01 | 7.3E+00 | 1.7E-01 | 8.6E+00 | 5.4E-01 | 1.5E-01 | 1.4E-01 | 1.2E-01 | 5.5E+00 | 7.9E-01 |
| rs4969266 | 3.1E-01 | 1.6E-01 | 3.2E-01 | 2.3E-01 | 2.4E-01 | 2.4E-01 | 2.5E-01 | 4.3E-01 | 3.2E-01 | 2.1E-01 | 2.1E-01 | 3.5E-01 | 2.7E-01 | 2.3E-01 | 1.0E-01 | 1.2E-01 | 2.1E-01 |
| rs4969429 | 3.0E-01 | 1.4E-01 | 1.8E-01 | 1.4E-01 | 1.8E-01 | 1.4E-01 | 1.8E-01 | 3.8E-01 | 1.6E-01 | 3.2E-01 | 3.0E-01 | 1.9E-01 | 1.3E-01 | 8.2E-02 | 1.1E-01 | 9.5E-02 | 1.0E-01 |
| rs7219896 | 4.0E+00 | 2.1E-01 | 5.1E-01 | 2.3E-01 | 4.0E-01 | 2.9E-01 | 4.6E-01 | 1.9E-01 | 1.4E+00 | 1.7E-01 | 6.4E-01 | 1.3E-01 | 1.2E-01 | 1.2E-01 | 1.8E-01 | 3.5E-01 | 1.5E-01 |
| rs7225574 | 3.3E+02 | 1.5E-01 | 4.4E-01 | 3.2E-01 | 7.9E-01 | 2.3E-01 | 5.5E-01 | 3.5E-01 | 1.1E+00 | 5.5E-01 | 2.4E+02 | 3.6E-01 | 1.6E-01 | 2.5E-01 | 1.5E-01 | 1.5E+00 | 1.3E-01 |
| rs11651724 | 6.7E-01 | 1.3E-01 | 2.3E-01 | 1.8E-01 | 2.6E-01 | 1.7E-01 | 2.4E-01 | 1.8E-01 | 1.4E-01 | 2.7E-01 | 5.1E-01 | 1.3E-01 | 1.3E-01 | 1.2E-01 | 8.5E-02 | 1.6E-01 | 1.0E-01 |
| rs4969444 | 3.1E+00 | 3.5E-01 | 4.8E-01 | 2.9E-01 | 4.1E-01 | 4.1E-01 | 4.4E-01 | 2.2E-01 | 8.1E-01 | 1.5E-01 | 5.7E-01 | 1.5E-01 | 1.3E-01 | 1.6E-01 | 2.2E-01 | 3.7E-01 | 1.6E-01 |
| rs2672886 | 2.6E+00 | 2.6E-01 | 3.8E+00 | 2.2E-01 | 4.7E+00 | 2.4E-01 | 3.9E+00 | 4.0E-01 | 1.6E-01 | 1.7E-01 | 1.2E+00 | 2.3E-01 | 1.4E-01 | 1.0E-01 | 2.9E-01 | 2.3E+00 | 1.1E-01 |
| rs2333990 | 2.8E-01 | 5.7E-01 | 1.7E-01 | 1.9E-01 | 1.6E-01 | 3.5E-01 | 1.7E-01 | 1.6E-01 | 1.8E-01 | 1.5E-01 | 2.6E-01 | 1.8E-01 | 1.4E-01 | 8.9E-02 | 1.7E-01 | 8.5E-02 | 1.1E-01 |
| rs2138125 | 1.8E+02 | 5.5E-01 | 4.8E+04 | 1.8E-01 | 1.6E+05 | 2.1E-01 | 8.4E+04 | 2.7E-01 | 5.9E-01 | 1.7E-01 | 1.0E+01 | 3.8E-01 | 3.6E-01 | 8.9E-02 | 3.2E-01 | 5.4E+04 | 2.4E-01 |
| rs2048753 | 1.6E+01 | 1.5E-01 | 2.1E+00 | 1.5E-01 | 1.3E+01 | 1.4E-01 | 2.4E+00 | 3.7E-01 | 1.7E-01 | 1.6E-01 | 3.4E+03 | 1.6E-01 | 1.4E-01 | 8.8E-02 | 1.1E-01 | 5.6E+00 | 1.1E-01 |
| rs2589133 | 1.7E+00 | 1.1E+00 | 4.3E+00 | 4.6E-01 | 2.3E+01 | 5.3E-01 | 6.0E+00 | 4.8E-01 | 3.9E-01 | 1.6E-01 | 2.2E+02 | 2.9E-01 | 4.3E-01 | 1.2E-01 | 3.3E-01 | 9.3E+00 | 2.7E-01 |
| rs7221948 | 2.5E-01 | 1.6E-01 | 1.7E-01 | 1.5E-01 | 1.8E-01 | 1.5E-01 | 1.7E-01 | 1.9E-01 | 5.3E-01 | 1.8E-01 | 2.0E-01 | 2.0E-01 | 2.9E-01 | 8.8E-02 | 1.2E-01 | 9.1E-02 | 3.0E-01 |
| rs7217174 | 2.1E-01 | 3.7E-01 | 1.9E-01 | 1.5E-01 | 1.7E-01 | 2.1E-01 | 1.8E-01 | 1.7E-01 | 1.7E-01 | 6.0E-01 | 2.4E-01 | 3.4E-01 | 1.4E-01 | 7.9E-02 | 5.4E-01 | 8.6E-02 | 1.4E-01 |
| rs2672901 | 2.3E-01 | 7.1E-01 | 6.0E-01 | 3.1E-01 | 1.8E+00 | 5.3E-01 | 7.8E-01 | 3.4E-01 | 2.5E-01 | 1.6E-01 | 3.7E-01 | 1.9E-01 | 4.1E-01 | 1.4E-01 | 2.9E-01 | 5.0E-01 | 2.2E-01 |
| rs7215379 | 2.7E-01 | 1.6E-01 | 2.7E-01 | 1.9E-01 | 5.3E-01 | 1.8E-01 | 2.9E-01 | 2.1E-01 | 2.4E-01 | 2.1E-01 | 3.8E-01 | 2.2E-01 | 1.7E-01 | 1.3E-01 | 1.1E-01 | 2.4E-01 | 1.5E-01 |
| rs2589143 | 2.3E-01 | 4.5E-01 | 1.0E+00 | 2.6E-01 | 7.7E-01 | 3.8E-01 | 8.9E-01 | 2.1E-01 | 4.2E-01 | 1.9E-01 | 2.4E-01 | 1.6E-01 | 5.1E-01 | 1.7E-01 | 1.4E-01 | 3.3E-01 | 3.3E-01 |
| rs746405 | 2.7E-01 | 5.0E-01 | 1.1E+00 | 2.8E-01 | 8.4E-01 | 3.5E-01 | 1.3E+00 | 5.5E-01 | 2.8E-01 | 4.2E-01 | 2.7E-01 | 1.7E-01 | 3.9E-01 | 2.6E-01 | 9.8E-02 | 3.3E-01 | 2.1E-01 |
| rs7219745 | 8.6E+00 | 3.4E-01 | 3.1E+02 | 1.4E-01 | 1.3E+02 | 1.8E-01 | 1.3E+02 | 4.2E-01 | 1.7E-01 | 4.1E-01 | 7.6E+00 | 2.2E-01 | 1.3E-01 | 7.7E-02 | 4.4E-01 | 4.9E+01 | 1.1E-01 |
| rs2589158 | 2.4E-01 | 1.6E-01 | 1.8E-01 | 4.7E-01 | 2.0E-01 | 3.6E-01 | 1.9E-01 | 3.1E-01 | 1.6E-01 | 4.4E-01 | 2.0E-01 | 1.5E-01 | 1.4E-01 | 7.7E-01 | 9.3E-02 | 1.0E-01 | 1.1E-01 |
| rs4969219 | 2.2E-01 | 6.4E-01 | 4.1E-01 | 4.8E-01 | 8.3E-01 | 6.2E-01 | 4.8E-01 | 2.3E-01 | 2.0E-01 | 6.4E-01 | 2.5E-01 | 1.6E-01 | 4.3E-01 | 5.6E-01 | 1.3E-01 | 2.7E-01 | 1.7E-01 |
| rs3829572 | 2.6E-01 | 3.4E-01 | 1.6E+00 | 2.3E-01 | 8.2E-01 | 2.8E-01 | 1.5E+00 | 2.8E-01 | 3.0E-01 | 3.2E-01 | 3.1E-01 | 1.4E-01 | 1.8E-01 | 1.7E-01 | 1.3E-01 | 3.9E-01 | 1.8E-01 |
| rs2589155 | 3.5E+00 | 3.2E-01 | 1.5E+01 | 1.4E-01 | 3.7E+01 | 1.6E-01 | 1.7E+01 | 2.9E-01 | 1.3E-01 | 2.3E-01 | 3.2E+00 | 1.7E-01 | 1.6E-01 | 7.4E-02 | 2.9E-01 | 1.2E+01 | 9.2E-02 |
| rs2589150 | 8.5E-01 | 1.7E-01 | 2.5E+00 | 1.3E-01 | 8.2E+00 | 1.4E-01 | 3.2E+00 | 6.6E-01 | 1.5E-01 | 1.9E-01 | 5.3E-01 | 1.5E-01 | 1.6E-01 | 7.5E-02 | 1.5E-01 | 2.2E+00 | 1.0E-01 |
| rs2589149 | 1.9E-01 | 1.2E-01 | 1.4E-01 | 1.4E-01 | 1.5E-01 | 1.3E-01 | 1.4E-01 | 2.6E-01 | 1.6E-01 | 1.7E-01 | 1.7E-01 | 1.2E-01 | 1.3E-01 | 9.4E-02 | 7.4E-02 | 7.8E-02 | 1.1E-01 |
| rs2672893 | 3.6E+00 | 2.1E-01 | 1.9E+01 | 1.3E-01 | 1.4E+01 | 1.3E-01 | 1.4E+01 | 2.7E-01 | 2.0E-01 | 2.0E-01 | 2.3E+00 | 1.6E-01 | 1.4E-01 | 7.5E-02 | 1.5E-01 | 6.6E+00 | 1.2E-01 |
| rs12943041 | 2.1E-01 | 1.3E-01 | 2.3E-01 | 1.5E-01 | 2.8E-01 | 1.4E-01 | 2.5E-01 | 2.8E-01 | 1.4E-01 | 1.6E-01 | 2.0E-01 | 1.5E-01 | 1.4E-01 | 1.1E-01 | 1.0E-01 | 1.4E-01 | 1.2E-01 |
| rs2672890 | 2.4E-01 | 2.3E-01 | 2.0E-01 | 1.4E-01 | 1.9E-01 | 1.7E-01 | 2.0E-01 | 1.8E-01 | 2.0E-01 | 1.8E-01 | 2.2E-01 | 2.9E-01 | 1.4E-01 | 9.1E-02 | 1.3E-01 | 1.0E-01 | 1.2E-01 |
| rs2589118 | 4.9E-01 | 2.1E-01 | 7.1E-01 | 1.5E-01 | 1.0E+00 | 1.5E-01 | 7.6E-01 | 1.9E-01 | 1.7E-01 | 3.1E-01 | 3.1E-01 | 2.1E-01 | 1.4E-01 | 9.0E-02 | 1.4E-01 | 3.2E-01 | 1.1E-01 |
| rs9912092 | 1.6E+00 | 1.7E-01 | 4.1E-01 | 1.5E-01 | 6.1E-01 | 1.5E-01 | 4.6E-01 | 5.4E-01 | 2.2E-01 | 3.2E-01 | 1.0E+00 | 1.8E-01 | 3.3E-01 | 1.1E-01 | 2.0E-01 | 3.0E-01 | 2.0E-01 |
| rs2589142 | 2.5E-01 | 1.3E-01 | 2.0E-01 | 1.4E-01 | 1.7E-01 | 1.3E-01 | 1.8E-01 | 2.1E-01 | 1.6E-01 | 2.9E-01 | 2.8E-01 | 1.4E-01 | 1.3E-01 | 8.8E-02 | 9.4E-02 | 8.7E-02 | 1.1E-01 |
| rs6565484 | 8.1E-01 | 1.8E-01 | 5.0E-01 | 1.5E-01 | 8.4E-01 | 1.5E-01 | 5.6E-01 | 5.6E-01 | 3.6E-01 | 2.4E-01 | 4.0E-01 | 1.5E-01 | 4.1E-01 | 8.3E-02 | 1.9E-01 | 3.2E-01 | 2.5E-01 |
| rs2289762 | 3.2E+00 | 1.6E-01 | 7.8E-01 | 1.4E-01 | 1.4E+00 | 1.4E-01 | 7.7E-01 | 1.1E+00 | 1.6E-01 | 2.2E-01 | 1.6E+00 | 1.4E-01 | 1.5E-01 | 8.2E-02 | 2.0E-01 | 7.6E-01 | 1.3E-01 |
| rs7219553 | 3.5E-01 | 2.3E-01 | 2.7E-01 | 2.3E-01 | 3.1E-01 | 2.4E-01 | 3.0E-01 | 2.4E-01 | 1.7E-01 | 1.7E-01 | 2.3E-01 | 1.5E-01 | 1.5E-01 | 1.2E-01 | 1.8E-01 | 1.4E-01 | 1.2E-01 |
| rs2289766 | 1.3E+01 | 2.5E-01 | 5.0E-01 | 1.5E-01 | 4.6E-01 | 2.1E-01 | 6.1E-01 | 1.8E-01 | 3.3E-01 | 1.1E+00 | 4.7E+00 | 1.9E-01 | 2.8E-01 | 1.2E-01 | 1.2E-01 | 8.6E-01 | 1.1E-01 |
| rs2280146 | 1.6E+00 | 2.8E-01 | 5.2E-01 | 1.5E-01 | 3.7E-01 | 2.6E-01 | 5.3E-01 | 1.7E-01 | 2.3E+00 | 2.5E-01 | 7.2E-01 | 1.4E-01 | 1.4E-01 | 1.1E-01 | 1.1E-01 | 4.1E-01 | 2.3E-01 |
| rs9898178 | 2.3E-01 | 1.5E-01 | 2.0E-01 | 1.7E-01 | 4.3E-01 | 1.5E-01 | 2.1E-01 | 1.8E-01 | 4.1E-01 | 1.5E-01 | 2.2E-01 | 1.3E-01 | 1.3E-01 | 9.2E-02 | 8.9E-02 | 1.1E-01 | 1.8E-01 |
| rs12951596 | 2.1E-01 | 1.4E-01 | 1.7E-01 | 1.3E-01 | 2.4E-01 | 1.3E-01 | 1.8E-01 | 2.0E-01 | 5.7E-01 | 1.5E-01 | 2.2E-01 | 1.3E-01 | 1.2E-01 | 7.5E-02 | 9.0E-02 | 9.6E-02 | 1.5E-01 |
| rs1468030 | 6.6E+00 | 2.6E+00 | 1.6E+00 | 3.4E-01 | 2.7E+00 | 7.3E-01 | 1.7E+00 | 2.8E+00 | 2.8E-01 | 5.5E-01 | 4.8E-01 | 2.1E-01 | 1.6E-01 | 1.4E-01 | 1.9E+00 | 6.1E-01 | 1.3E-01 |
| rs7220348 | 7.7E-01 | 9.1E+03 | 7.9E+00 | 4.4E-01 | 1.1E+00 | 1.1E+01 | 3.0E+00 | 8.7E-01 | 3.9E-01 | 6.5E-01 | 3.7E-01 | 1.9E-01 | 1.7E-01 | 1.2E-01 | 1.1E+03 | 2.8E-01 | 1.3E-01 |
| rs9897968 | 5.0E-01 | 1.6E-01 | 1.7E-01 | 1.4E-01 | 1.8E-01 | 1.5E-01 | 1.7E-01 | 2.3E-01 | 1.8E-01 | 1.6E-01 | 5.0E-01 | 1.4E-01 | 1.4E-01 | 8.6E-02 | 1.1E-01 | 1.2E-01 | 1.1E-01 |
| rs9901846 | 2.7E-01 | 2.1E-01 | 4.0E-01 | 2.3E-01 | 3.1E-01 | 2.4E-01 | 3.2E-01 | 1.8E-01 | 4.7E-01 | 4.3E-01 | 2.5E-01 | 1.7E-01 | 1.8E-01 | 1.6E-01 | 1.4E-01 | 1.2E-01 | 1.1E-01 |
| rs9908270 | 2.4E-01 | 3.6E-01 | 2.5E-01 | 3.6E-01 | 2.1E-01 | 7.3E-01 | 2.2E-01 | 2.4E-01 | 5.1E-01 | 3.1E-01 | 3.0E-01 | 1.4E-01 | 2.3E-01 | 1.5E-01 | 1.3E-01 | 1.0E-01 | 1.1E-01 |
| rs2271602 | 1.1E+00 | 6.9E-01 | 8.1E-01 | 2.9E-01 | 6.8E-01 | 4.3E-01 | 7.8E-01 | 4.1E-01 | 5.8E-01 | 8.6E-01 | 5.2E-01 | 1.5E-01 | 1.3E-01 | 1.3E-01 | 1.4E+00 | 3.0E-01 | 1.5E-01 |
| rs2271608 | 4.8E-01 | 1.8E-01 | 1.7E-01 | 1.4E-01 | 1.9E-01 | 1.6E-01 | 1.8E-01 | 6.9E-01 | 2.6E-01 | 3.0E-01 | 3.9E-01 | 2.4E-01 | 1.5E-01 | 8.4E-02 | 2.4E-01 | 1.2E-01 | 1.1E-01 |
| rs4969227 | 3.9E-01 | 1.3E+01 | 1.2E+01 | 1.4E-01 | 2.3E-01 | 1.9E-01 | 2.5E+00 | 2.1E-01 | 1.6E-01 | 4.0E+01 | 2.1E-01 | 1.5E-01 | 2.6E-01 | 8.4E-02 | 1.7E+01 | 1.3E-01 | 2.0E-01 |
| rs11655629 | 2.7E-01 | 1.1E-01 | 1.9E-01 | 9.9E-02 | 1.9E-01 | 1.0E-01 | 1.8E-01 | 7.2E-01 | 1.8E+00 | 2.2E-01 | 2.2E-01 | 2.0E-01 | 3.8E-01 | 7.5E-02 | 1.1E-01 | 9.8E-02 | 5.1E-01 |
| rs1877926 | 1.0E+00 | 3.8E-01 | 3.3E-01 | 1.7E-01 | 3.4E-01 | 2.3E-01 | 3.3E-01 | 4.3E-01 | 1.6E-01 | 9.0E-01 | 4.5E-01 | 1.6E-01 | 1.6E-01 | 8.9E-02 | 3.9E-01 | 1.8E-01 | 1.1E-01 |
| rs9911223 | 2.2E-01 | 1.9E-01 | 2.6E-01 | 1.3E-01 | 1.8E-01 | 1.5E-01 | 2.2E-01 | 3.9E-01 | 1.6E-01 | 1.8E-01 | 2.1E-01 | 1.8E-01 | 2.1E-01 | 8.7E-02 | 9.1E-02 | 9.5E-02 | 1.1E-01 |
| rs7219318 | 2.5E-01 | 3.2E-01 | 2.1E-01 | 1.5E-01 | 2.0E-01 | 1.6E-01 | 2.0E-01 | 1.1E+00 | 6.5E-01 | 1.9E-01 | 3.2E-01 | 1.8E-01 | 1.7E-01 | 1.0E-01 | 1.1E-01 | 1.1E-01 | 1.4E-01 |
| rs6565498 | 4.4E-01 | 1.5E-01 | 2.3E-01 | 1.2E-01 | 1.8E-01 | 1.5E-01 | 2.3E-01 | 3.1E-01 | 1.3E+00 | 1.6E-01 | 2.3E-01 | 1.3E-01 | 1.3E-01 | 7.5E-02 | 1.1E-01 | 1.2E-01 | 2.1E-01 |
| rs2878052 | 6.3E-01 | 4.3E-01 | 2.3E-01 | 2.2E-01 | 2.5E-01 | 3.1E-01 | 2.4E-01 | 5.8E-01 | 3.8E-01 | 1.6E-01 | 5.4E-01 | 1.6E-01 | 1.4E-01 | 9.2E-02 | 3.9E-01 | 1.5E-01 | 1.5E-01 |
| rs2271612 | 1.1E+00 | 2.6E+05 | 1.2E+00 | 1.7E+00 | 4.0E-01 | 1.2E+03 | 8.5E-01 | 2.5E-01 | 1.6E-01 | 3.2E-01 | 3.2E-01 | 1.5E-01 | 1.7E-01 | 4.0E-01 | 1.1E+02 | 3.4E-01 | 1.2E-01 |
| rs7209380 | 8.4E-01 | 3.9E-01 | 3.4E-01 | 6.9E-01 | 1.4E+00 | 7.4E-01 | 5.6E-01 | 1.7E+00 | 2.1E-01 | 1.5E-01 | 3.8E+00 | 1.3E-01 | 2.9E-01 | 1.9E-01 | 4.4E-01 | 6.5E-01 | 2.3E-01 |
| rs9907231 | 2.7E-01 | 1.6E-01 | 2.2E-01 | 2.4E-01 | 2.2E-01 | 1.8E-01 | 2.1E-01 | 9.1E-01 | 2.8E-01 | 6.5E-01 | 2.4E-01 | 4.6E-01 | 4.7E-01 | 2.9E-01 | 2.1E-01 | 1.1E-01 | 3.1E-01 |
| rs6420481 | 2.6E-01 | 1.2E+02 | 1.5E+00 | 2.0E-01 | 2.0E-01 | 2.5E-01 | 3.5E-01 | 2.8E-01 | 1.7E-01 | 4.5E-01 | 6.7E-01 | 3.6E-01 | 1.6E+00 | 1.2E-01 | 3.5E-01 | 1.1E-01 | 3.8E-01 |
| rs1468027 | 2.6E+00 | 1.9E-01 | 8.7E-01 | 1.4E-01 | 8.4E-01 | 1.7E-01 | 8.8E-01 | 1.7E-01 | 1.5E-01 | 2.0E-01 | 2.7E+00 | 3.1E-01 | 2.1E-01 | 9.5E-02 | 1.4E-01 | 5.9E-01 | 1.2E-01 |
| rs7225525 | 5.5E+00 | 2.4E-01 | 2.3E-01 | 1.5E-01 | 2.2E-01 | 1.4E-01 | 2.0E-01 | 1.9E-01 | 1.7E-01 | 3.2E-01 | 6.4E+00 | 1.2E+00 | 5.8E-01 | 8.9E-02 | 2.6E-01 | 1.5E-01 | 1.7E-01 |
| rs7224748 | 3.3E-01 | 1.5E+00 | 3.0E-01 | 2.8E-01 | 2.9E-01 | 1.6E-01 | 2.0E-01 | 3.3E-01 | 3.9E-01 | 2.9E-01 | 5.4E+00 | 6.1E-01 | 3.8E+01 | 1.5E-01 | 1.7E-01 | 1.7E-01 | 3.2E+00 |
| rs9908043 | 5.0E-01 | 1.8E-01 | 3.9E-01 | 1.1E-01 | 2.2E-01 | 1.5E-01 | 3.4E-01 | 1.7E-01 | 9.0E-01 | 2.3E-01 | 3.4E-01 | 1.3E-01 | 1.6E-01 | 6.7E-02 | 1.4E-01 | 1.1E-01 | 2.2E-01 |
| rs3751934 | 4.4E-01 | 1.5E-01 | 4.0E-01 | 6.0E-01 | 2.0E-01 | 2.8E-01 | 2.6E-01 | 2.0E-01 | 2.8E-01 | 4.5E-01 | 2.4E-01 | 1.8E-01 | 1.2E+00 | 3.4E-01 | 9.2E-02 | 1.1E-01 | 5.0E-01 |
| rs3751932 | 2.4E-01 | 1.4E-01 | 3.0E-01 | 1.2E-01 | 1.8E-01 | 1.1E-01 | 2.2E-01 | 3.6E+00 | 1.5E+00 | 9.8E-01 | 2.0E-01 | 3.6E-01 | 3.7E-01 | 8.8E-02 | 2.5E-01 | 1.0E-01 | 1.0E+00 |
| rs1062935 | 2.8E-01 | 2.1E-01 | 4.1E-01 | 1.4E-01 | 2.2E-01 | 1.5E-01 | 3.1E-01 | 1.9E-01 | 2.1E-01 | 2.1E-01 | 3.7E-01 | 2.7E-01 | 1.3E-01 | 8.6E-02 | 1.5E-01 | 1.1E-01 | 1.1E-01 |
| rs6565507 | 3.7E-01 | 2.4E-01 | 7.5E-01 | 1.5E-01 | 2.0E-01 | 1.5E-01 | 3.7E-01 | 2.0E-01 | 4.3E+00 | 2.3E+00 | 4.9E-01 | 2.8E-01 | 4.2E-01 | 9.0E-02 | 2.7E-01 | 1.2E-01 | 1.9E+00 |
| rs7502124 | 2.5E-01 | 3.6E-01 | 5.8E-01 | 1.6E-01 | 2.0E-01 | 2.5E-01 | 3.0E-01 | 5.3E-01 | 3.2E+00 | 1.1E+00 | 5.1E-01 | 3.2E-01 | 5.9E-01 | 9.9E-02 | 2.6E-01 | 1.0E-01 | 2.1E+00 |
| rs6565508 | 2.6E-01 | 2.0E-01 | 2.9E-01 | 1.3E-01 | 2.1E-01 | 1.3E-01 | 2.0E-01 | 3.4E-01 | 2.2E-01 | 2.1E-01 | 5.5E-01 | 1.4E-01 | 1.6E-01 | 7.8E-02 | 1.2E-01 | 1.1E-01 | 1.6E-01 |
| rs11653897 | 2.9E-01 | 1.9E-01 | 3.5E-01 | 1.8E+00 | 2.1E-01 | 9.1E-01 | 2.6E-01 | 2.3E-01 | 3.0E-01 | 5.6E-01 | 2.6E-01 | 1.4E+00 | 1.7E+00 | 9.2E-01 | 9.5E-02 | 1.1E-01 | 8.3E-01 |
| rs1399571 | 3.1E-01 | 4.8E-01 | 2.6E-01 | 2.3E-01 | 2.6E-01 | 4.6E-01 | 2.1E-01 | 2.7E-01 | 1.5E-01 | 1.9E-01 | 2.6E+00 | 2.1E-01 | 1.6E-01 | 2.0E-01 | 1.1E-01 | 1.4E-01 | 1.3E-01 |
| rs6565511 | 5.5E-01 | 1.2E-01 | 1.8E-01 | 1.1E-01 | 1.5E-01 | 1.1E-01 | 1.5E-01 | 5.4E-01 | 2.1E+02 | 1.0E+01 | 1.7E-01 | 1.4E-01 | 4.2E-01 | 7.1E-02 | 1.6E-01 | 8.1E-02 | 3.5E+01 |
| rs7219486 | 2.7E-01 | 1.9E-01 | 2.1E-01 | 1.4E-01 | 2.0E-01 | 1.7E-01 | 1.8E-01 | 2.9E-01 | 1.6E+00 | 3.1E-01 | 1.1E+00 | 1.3E-01 | 5.0E-01 | 8.8E-02 | 1.1E-01 | 1.0E-01 | 1.1E+00 |
| rs4969331 | 2.8E-01 | 1.4E-01 | 6.3E-01 | 6.8E-01 | 2.5E-01 | 2.7E-01 | 3.6E-01 | 3.9E-01 | 2.6E-01 | 6.6E+01 | 5.6E-01 | 1.1E+00 | 4.3E+00 | 9.0E-01 | 2.5E-01 | 1.1E-01 | 2.2E+00 |
| rs8081168 | 6.6E-01 | 2.9E-01 | 2.3E-01 | 1.3E+00 | 6.3E-01 | 6.2E-01 | 3.0E-01 | 3.7E-01 | 1.9E-01 | 7.6E-01 | 1.9E+00 | 1.7E-01 | 5.7E-01 | 4.0E-01 | 1.2E-01 | 3.5E-01 | 2.6E-01 |
| rs7219221 | 2.7E+00 | 3.1E-01 | 1.1E+00 | 1.7E-01 | 1.1E+00 | 2.0E-01 | 1.0E+00 | 2.0E+00 | 6.7E-01 | 1.7E-01 | 6.0E-01 | 2.7E-01 | 1.4E-01 | 7.6E-02 | 4.9E-01 | 6.6E-01 | 2.1E-01 |
| rs7225916 | 8.6E-01 | 1.6E-01 | 3.0E-01 | 1.6E-01 | 2.5E-01 | 1.7E-01 | 2.7E-01 | 2.3E-01 | 2.2E-01 | 3.0E-01 | 4.7E-01 | 1.5E-01 | 1.8E-01 | 1.0E-01 | 1.3E-01 | 1.6E-01 | 1.3E-01 |
| rs7502321 | 5.0E-01 | 1.4E-01 | 2.6E-01 | 1.4E-01 | 2.0E-01 | 1.4E-01 | 2.2E-01 | 2.8E-01 | 1.5E-01 | 1.5E-01 | 2.8E-01 | 1.7E-01 | 1.4E-01 | 8.4E-02 | 9.2E-02 | 1.2E-01 | 1.1E-01 |
| rs8072124 | 1.4E+00 | 2.4E-01 | 5.9E-01 | 1.5E-01 | 7.2E-01 | 1.6E-01 | 5.8E-01 | 4.4E-01 | 1.8E-01 | 1.8E-01 | 3.0E+00 | 3.9E-01 | 1.7E-01 | 8.9E-02 | 2.4E-01 | 4.3E-01 | 1.3E-01 |
| rs4969349 | 2.5E-01 | 2.2E-01 | 3.2E-01 | 2.6E-01 | 6.7E-01 | 2.8E-01 | 4.8E-01 | 1.9E-01 | 1.2E+00 | 2.1E-01 | 5.9E-01 | 1.7E-01 | 1.3E+00 | 2.1E-01 | 9.4E-02 | 3.2E-01 | 5.7E-01 |
| rs4969355 | 2.9E-01 | 5.1E-01 | 3.6E-01 | 3.0E-01 | 3.8E-01 | 4.0E-01 | 3.8E-01 | 2.7E-01 | 4.2E-01 | 3.8E-01 | 2.4E-01 | 1.8E-01 | 1.4E-01 | 1.3E-01 | 4.1E-01 | 1.3E-01 | 1.5E-01 |
| rs9906253 | 1.9E-01 | 4.4E-01 | 4.9E-01 | 4.1E-01 | 6.8E-01 | 4.4E-01 | 5.5E-01 | 1.7E-01 | 4.1E-01 | 1.5E-01 | 1.9E-01 | 1.3E-01 | 1.7E-01 | 1.8E-01 | 1.6E-01 | 2.0E-01 | 1.6E-01 |
| rs12051877 | 2.4E+00 | 3.6E-01 | 3.9E+01 | 1.7E-01 | 4.7E-01 | 2.5E-01 | 7.0E+00 | 2.2E+00 | 1.6E-01 | 2.1E+03 | 2.4E-01 | 6.4E-01 | 3.2E-01 | 9.0E-02 | 3.5E+03 | 2.5E-01 | 2.7E-01 |
| rs11869351 | 4.9E+00 | 1.2E+00 | 3.7E+00 | 1.7E-01 | 2.4E+00 | 2.9E-01 | 3.6E+00 | 2.3E-01 | 1.4E-01 | 3.0E-01 | 1.6E+00 | 8.2E-01 | 1.3E-01 | 8.3E-02 | 9.7E-01 | 1.6E+00 | 1.0E-01 |
| rs8079626 | 2.4E-01 | 5.1E-01 | 3.9E-01 | 3.0E-01 | 5.1E-01 | 3.3E-01 | 4.2E-01 | 2.6E-01 | 4.1E-01 | 1.9E-01 | 2.7E-01 | 1.4E-01 | 2.9E+00 | 1.3E-01 | 3.5E-01 | 2.3E-01 | 7.1E-01 |
| rs12945231 | 2.0E-01 | 1.7E-01 | 1.8E-01 | 1.4E-01 | 1.6E-01 | 1.8E-01 | 1.8E-01 | 2.9E-01 | 2.2E-01 | 1.4E-01 | 1.7E-01 | 1.5E-01 | 1.2E-01 | 1.0E-01 | 8.5E-02 | 8.5E-02 | 1.1E-01 |
| rs4969367 | 1.3E+01 | 1.5E-01 | 3.9E+00 | 1.3E-01 | 4.0E+00 | 1.3E-01 | 5.4E+00 | 3.5E-01 | 6.8E-01 | 2.8E-01 | 3.4E+00 | 2.7E+00 | 1.5E-01 | 9.6E-02 | 2.0E-01 | 3.0E+00 | 1.9E-01 |
| rs9901648 | 4.6E+01 | 1.6E+01 | 9.0E+01 | 9.1E+00 | 2.0E+03 | 1.9E+01 | 3.7E+02 | 2.3E-01 | 3.2E-01 | 1.6E-01 | 2.2E+02 | 1.9E-01 | 9.3E-01 | 2.4E+00 | 1.9E+00 | 4.5E+02 | 3.2E-01 |
