# Supplementary material for: Allele-Specific Down-Regulation of RPTOR Expression Induced by Retinoids Contributes to Climate Adaptations
Source: PLoS Genet. 2010 Oct 28;6(10):e1001178. doi: 10.1371/journal.pgen.1001178 (PMC2965758; doi:10.1371/journal.pgen.1001178)
Supplement: Table S3 — Bayes Factors empirical p values for the RPTOR SNPs in the HGDP panel. (0.55 MB DOC) [file pgen.1001178.s007.doc]

Table S3: Bayes Factors empirical p values for the *RPTOR* SNPs in the HGDP panal (in bold, p<0.05 or for rs11868112).

| SNP | Latitude | Minimum T (summer) | Minimum T (winter) | Maximum T (summer) | Maximum T (winter) | Mean T (summer) | Mean T (winter) | Precipitation rate (summer) | Precipitation rate (winter) | Short-Wave radiation (summer) | Short-Wave radiation (winter) | Relative humidity (summer) | Relative humidity (winter) | PC1 (summer) | PC2 (summer) | PC1 (winter) | PC2 (winter) |
| --- | --- | --- | --- | --- | --- | --- | --- | --- | --- | --- | --- | --- | --- | --- | --- | --- | --- |
| rs4074302 | 0.88 | 0.21 | 0.80 | 0.21 | 0.32 | 0.23 | 0.58 | 0.91 | 0.59 | 0.96 | 0.52 | 0.93 | 0.16 | 0.28 | 0.50 | 0.40 | 0.26 |
| rs8071962 | 0.25 | **0.04** | 0.09 | 0.06 | 0.06 | **0.04** | 0.08 | 0.14 | 0.22 | 0.86 | 0.15 | 0.16 | 0.31 | 0.10 | 0.10 | 0.08 | 0.19 |
| rs4890025 | 0.15 | 0.66 | 0.24 | 0.25 | 0.16 | 0.36 | 0.21 | 0.52 | 0.88 | 0.11 | 0.08 | 0.27 | 0.60 | 0.24 | 0.65 | 0.16 | 0.80 |
| rs7503219 | 0.09 | 0.81 | 0.34 | 0.71 | 0.36 | 0.77 | 0.40 | 0.81 | 0.05 | 0.48 | 0.21 | 0.83 | 0.77 | 0.68 | 0.75 | 0.27 | 0.26 |
| rs10931 | 0.54 | 0.54 | 0.46 | 0.15 | 0.16 | 0.24 | 0.32 | 0.50 | 0.05 | 0.48 | 0.14 | 0.43 | 0.38 | 0.21 | 0.86 | 0.26 | 0.12 |
| rs12943620 | 0.79 | 0.47 | 0.54 | 0.28 | 0.46 | 0.28 | 0.54 | 0.12 | 0.30 | 0.64 | 0.73 | 0.09 | 0.76 | 0.18 | 0.59 | 0.53 | 0.46 |
| rs11869626 | 0.37 | 0.95 | 0.45 | 0.89 | 0.45 | 0.90 | 0.52 | 0.17 | 0.34 | 0.60 | 0.46 | 0.74 | 0.93 | 0.78 | 0.97 | 0.44 | 0.55 |
| rs11657796 | 0.70 | 0.84 | 0.79 | 0.73 | 0.72 | 0.77 | 0.81 | 0.57 | 0.40 | 0.36 | 0.49 | 0.75 | 0.90 | 0.78 | 0.85 | 0.76 | 0.60 |
| rs7213201 | 0.27 | 0.79 | 0.41 | 0.69 | 0.47 | 0.73 | 0.48 | 0.32 | 0.25 | 0.41 | 0.38 | 0.46 | 0.89 | 0.63 | 0.53 | 0.34 | 0.54 |
| rs4453556 | 0.56 | 0.97 | 0.59 | 0.58 | 0.92 | 0.67 | 0.80 | 0.79 | 0.57 | 0.55 | 0.84 | 0.54 | 0.39 | 0.51 | 0.92 | 0.91 | 0.43 |
| rs4561525 | 0.08 | 0.64 | 0.22 | 0.67 | 0.07 | 0.65 | 0.12 | 0.13 | 0.66 | 0.91 | **0.04** | 0.27 | 0.76 | 0.52 | 0.39 | 0.07 | 0.89 |
| rs11650988 | 0.96 | 0.68 | 0.32 | 0.75 | 0.33 | 0.86 | 0.33 | 0.52 | 0.37 | 0.44 | 0.93 | 0.42 | 0.80 | 0.64 | 0.39 | 0.37 | 0.49 |
| rs7212223 | 0.09 | 0.65 | 0.20 | 0.93 | 0.16 | 0.73 | 0.18 | 0.05 | 0.22 | 0.45 | **0.03** | 0.62 | 0.70 | 0.83 | 0.21 | 0.11 | 0.90 |
| rs7216808 | 0.39 | 0.91 | 0.51 | 0.62 | 0.59 | 0.72 | 0.56 | 0.61 | 0.31 | 0.69 | 0.46 | 0.99 | 0.98 | 0.63 | 0.83 | 0.46 | 0.71 |
| rs12601738 | **0.01** | 0.62 | **0.03** | 0.65 | **0.01** | 0.73 | **0.02** | 0.14 | 0.11 | 0.47 | **0.01** | 0.07 | 0.92 | 0.37 | 0.29 | **0.01** | 0.31 |
| **rs11868112** | **0.02** | 0.79 | **0.01** | 0.19 | **<0.01** | 0.53 | **0.01** | 0.06 | **0.04** | 0.51 | **0.04** | 0.19 | 0.61 | 0.08 | 0.33 | **<0.01** | 0.21 |
| rs7224003 | 0.74 | 0.93 | 0.30 | 0.66 | 0.30 | 0.87 | 0.31 | 0.79 | 0.46 | 0.49 | 0.82 | 0.33 | 0.81 | 0.62 | 0.64 | 0.36 | 0.54 |
| rs4073061 | 0.50 | 0.05 | 0.23 | **0.04** | 0.12 | **0.04** | 0.16 | 0.83 | 0.44 | 0.21 | 0.36 | 0.74 | 0.35 | 0.07 | 0.28 | 0.14 | 0.34 |
| rs4890037 | 0.26 | 0.70 | 0.33 | 0.75 | 0.24 | 0.79 | 0.27 | 0.07 | 0.08 | 0.84 | 0.17 | 0.05 | 0.57 | 0.59 | 0.28 | 0.23 | 0.27 |
| rs4889856 | 0.08 | 0.70 | **0.04** | 0.67 | 0.06 | 0.66 | **0.04** | 0.29 | 0.07 | 0.85 | 0.20 | 0.18 | 0.21 | 0.54 | 0.91 | 0.05 | **0.04** |
| rs3923514 | 0.99 | 0.58 | 0.98 | 0.59 | 0.77 | 0.56 | 0.94 | 0.98 | 0.94 | 0.99 | 0.55 | 0.97 | 0.29 | 0.67 | 0.86 | 0.75 | 0.59 |
| rs8075710 | 0.62 | 0.11 | 0.11 | 0.10 | 0.20 | 0.11 | 0.16 | 0.35 | 0.84 | 0.08 | 0.42 | 0.59 | 0.64 | 0.23 | 0.33 | 0.22 | 0.80 |
| rs4627412 | 0.52 | 0.80 | 0.73 | 0.88 | 0.58 | 0.76 | 0.71 | 0.92 | 0.54 | 0.69 | 0.50 | 0.82 | 0.64 | 0.92 | 0.74 | 0.54 | 0.89 |
| rs9911978 | 0.63 | 0.77 | 0.74 | 0.83 | 0.41 | 0.75 | 0.68 | 0.58 | 0.52 | 0.74 | 0.65 | 0.76 | 0.58 | 0.79 | 0.75 | 0.46 | 0.86 |
| rs4890042 | 0.34 | 0.99 | 0.48 | 0.64 | 0.20 | 0.94 | 0.42 | 0.96 | 0.21 | 0.26 | 0.13 | 0.29 | 0.13 | 0.50 | 0.77 | 0.17 | 0.15 |
| rs12937147 | 0.21 | 0.78 | 0.08 | 0.74 | **0.03** | 0.70 | 0.06 | 0.54 | 0.78 | 0.99 | 0.07 | 0.32 | 0.41 | 0.60 | 0.72 | **0.04** | 0.90 |
| rs7209040 | 0.53 | 0.94 | 0.23 | 0.83 | 0.16 | 0.82 | 0.14 | 0.23 | 0.85 | 0.85 | 0.43 | 0.24 | 0.75 | 0.65 | 0.41 | 0.27 | 0.62 |
| rs11871623 | 0.57 | 0.55 | 0.16 | 0.42 | 0.22 | 0.45 | 0.14 | 0.70 | 0.61 | 0.67 | 0.55 | 0.33 | 0.66 | 0.42 | 0.75 | 0.22 | 0.60 |
| rs7226296 | 0.55 | 0.94 | **0.04** | 0.98 | 0.02 | 0.97 | **0.03** | 0.66 | 0.90 | 0.73 | 0.33 | 0.43 | 0.94 | 0.96 | 0.82 | **0.03** | 0.95 |
| rs7215496 | 0.15 | 0.91 | 0.05 | 0.47 | **0.04** | 0.56 | **0.04** | 0.52 | 0.29 | 0.76 | 0.19 | 0.19 | 0.92 | 0.36 | 0.66 | **0.04** | 0.52 |
| rs4889863 | 0.66 | 0.77 | 0.79 | 0.83 | 0.66 | 0.86 | 0.77 | 0.46 | 0.95 | 0.53 | 0.32 | 0.64 | 0.47 | 0.92 | 0.43 | 0.66 | 0.83 |
| rs12951309 | 0.08 | 0.65 | 0.09 | 0.56 | **0.04** | 0.68 | 0.06 | 0.46 | 0.68 | 0.58 | **0.04** | 0.21 | 0.39 | 0.41 | 0.45 | **0.04** | 0.85 |
| rs12949279 | 0.17 | 0.91 | 0.28 | 0.69 | 0.14 | 0.80 | 0.21 | 0.91 | 0.75 | 0.81 | 0.07 | 0.73 | 0.18 | 0.73 | 0.94 | 0.13 | 0.63 |
| rs7210046 | 0.27 | 0.09 | 0.26 | 0.15 | 0.24 | 0.12 | 0.25 | 0.12 | 0.23 | 0.21 | 0.31 | 0.69 | 0.35 | 0.20 | 0.42 | 0.25 | 0.22 |
| rs11653499 | 0.36 | 0.95 | 0.26 | 0.88 | 0.12 | 0.97 | 0.15 | 0.82 | 0.86 | 0.36 | 0.27 | 0.81 | 0.30 | 0.89 | 0.76 | 0.14 | 0.47 |
| rs4890055 | 0.83 | 0.38 | 0.99 | 0.74 | 0.75 | 0.93 | 0.98 | 0.75 | 0.67 | 0.18 | 0.17 | 0.10 | 0.35 | 0.57 | 0.25 | 0.72 | 0.61 |
| rs9890502 | 0.22 | 0.58 | 0.25 | 0.41 | 0.10 | 0.55 | 0.16 | 0.86 | 0.86 | 0.68 | 0.15 | 0.94 | 0.52 | 0.50 | 0.74 | 0.11 | 0.78 |
| rs7503807 | 0.07 | 0.98 | 0.13 | 0.84 | 0.07 | 0.98 | 0.10 | 0.60 | 0.28 | 0.65 | 0.06 | 0.56 | 0.16 | 0.93 | 0.89 | 0.05 | 0.65 |
| rs9903842 | 0.17 | 0.21 | **0.02** | 0.32 | **0.03** | 0.30 | **0.02** | 0.32 | 0.29 | 0.29 | 0.22 | 0.63 | 0.44 | 0.35 | 0.10 | 0.05 | 0.34 |
| rs901065 | 0.11 | 0.59 | 0.49 | 0.66 | 0.28 | 0.36 | 0.42 | 0.41 | 0.14 | 0.56 | 0.05 | 0.53 | 0.81 | 0.30 | 0.99 | 0.23 | 0.55 |
| rs8071015 | 0.14 | 0.59 | 0.15 | 0.41 | 0.11 | 0.49 | 0.13 | 0.33 | 0.86 | 0.52 | 0.05 | 0.66 | 0.35 | 0.55 | 0.73 | 0.12 | 0.68 |
| rs999977 | 0.96 | 0.29 | 0.67 | 0.22 | 0.84 | 0.19 | 0.70 | 0.82 | 0.65 | 0.83 | 1.00 | 0.96 | 0.51 | 0.32 | 0.39 | 0.79 | 0.57 |
| rs12940622 | 0.06 | 0.58 | 0.16 | 0.40 | 0.09 | 0.45 | 0.11 | 0.83 | 0.43 | 0.65 | **0.02** | 0.92 | 0.38 | 0.44 | 0.65 | 0.06 | 0.96 |
| rs9900506 | 0.40 | 0.66 | 0.54 | 0.72 | 0.55 | 0.70 | 0.58 | 0.92 | 0.37 | 0.88 | 0.93 | 0.27 | 0.44 | 0.70 | 0.79 | 0.58 | 0.46 |
| rs7212142 | 0.06 | 0.63 | 0.05 | 0.99 | 0.11 | 0.99 | 0.05 | 0.64 | 0.41 | 0.44 | 0.31 | 0.09 | 0.84 | 0.94 | 0.40 | 0.10 | 0.47 |
| rs12939076 | **0.02** | 0.64 | 0.08 | 0.53 | 0.07 | 0.57 | 0.09 | 0.34 | **0.02** | 0.58 | **0.02** | 0.07 | 0.47 | 0.32 | 0.40 | 0.06 | 0.13 |
| rs9906493 | 0.78 | 0.11 | 0.33 | **0.02** | 0.09 | 0.05 | 0.16 | 0.30 | **0.02** | 0.28 | 0.09 | 0.43 | **0.01** | **0.02** | 0.66 | 0.16 | **0.01** |
| rs4889782 | **0.04** | 0.76 | 0.10 | 0.93 | 0.09 | 0.85 | 0.08 | 0.89 | 0.91 | 0.34 | 0.05 | 0.74 | 0.99 | 0.90 | 0.94 | 0.08 | 0.98 |
| rs9889835 | 0.23 | 0.13 | 0.35 | 0.12 | 0.24 | 0.11 | 0.28 | 0.51 | 0.63 | 0.27 | 0.29 | 0.62 | 0.49 | 0.20 | 0.37 | 0.22 | 0.62 |
| rs1485330 | **0.01** | 0.78 | 0.05 | 0.56 | **0.03** | 0.57 | 0.05 | 0.51 | 0.06 | 0.82 | **0.03** | 0.16 | 0.52 | 0.47 | 0.56 | **0.03** | 0.17 |
| rs6565472 | 0.21 | 0.46 | 0.12 | 0.39 | 0.07 | 0.57 | 0.10 | 0.72 | 0.93 | 0.42 | 0.07 | 0.36 | 0.28 | 0.36 | 0.46 | 0.09 | 0.53 |
| rs7217223 | 0.28 | 0.55 | 0.30 | 0.66 | 0.09 | 0.73 | 0.18 | 0.52 | 0.81 | 0.50 | 0.07 | 0.56 | 0.23 | 0.59 | 0.43 | 0.09 | 0.38 |
| rs4889875 | 0.58 | 0.74 | 0.59 | 0.91 | 0.63 | 0.82 | 0.62 | 0.89 | 0.16 | 0.80 | 0.81 | 0.33 | 0.76 | 0.76 | 0.72 | 0.56 | 0.29 |
| rs9901366 | 0.83 | 0.10 | 0.62 | 0.08 | 0.80 | 0.08 | 0.64 | 0.36 | 0.44 | 0.73 | 0.89 | 0.38 | 0.34 | 0.06 | 0.53 | 0.71 | 0.45 |
| rs7501659 | 0.79 | 0.46 | 1.00 | 0.99 | 0.82 | 0.99 | 0.98 | 0.88 | 0.15 | 0.46 | 0.56 | 0.22 | 0.73 | 0.96 | 0.43 | 0.77 | 0.58 |
| rs9906827 | 0.23 | 0.97 | 0.22 | 0.44 | 0.37 | 0.63 | 0.31 | 0.33 | 0.06 | 0.64 | 0.55 | 0.06 | 0.16 | 0.33 | 0.64 | 0.34 | 0.11 |
| rs9902891 | 0.41 | 0.46 | 0.28 | 0.54 | 0.30 | 0.48 | 0.25 | 0.68 | 0.72 | 0.59 | 0.51 | 0.32 | 0.77 | 0.57 | 0.53 | 0.30 | 0.83 |
| rs7208502 | 0.16 | 0.91 | 0.17 | 0.33 | 0.31 | 0.49 | 0.25 | 0.40 | 0.11 | 0.66 | 0.43 | **0.04** | 0.23 | 0.21 | 0.60 | 0.27 | 0.11 |
| rs12948054 | 0.11 | 0.58 | 0.69 | 0.36 | 0.47 | 0.35 | 0.56 | 0.86 | 0.62 | 0.90 | 0.13 | 0.89 | 0.45 | 0.36 | 0.84 | 0.34 | 0.80 |
| rs4062178 | 0.97 | 0.93 | 0.96 | 0.24 | 0.99 | 0.35 | 0.99 | 0.41 | 0.73 | 0.71 | 0.98 | 0.06 | 0.25 | 0.15 | 0.90 | 0.99 | 0.34 |
| rs1564868 | 0.23 | 0.77 | 0.25 | 0.63 | 0.37 | 0.69 | 0.31 | 0.49 | 0.13 | 0.93 | 0.38 | 0.26 | 0.37 | 0.52 | 0.69 | 0.28 | 0.22 |
| rs7211818 | 0.86 | 0.67 | 0.97 | 0.29 | 0.50 | 0.54 | 0.79 | 0.96 | 0.46 | 0.84 | 0.39 | 0.31 | 0.21 | 0.35 | 0.95 | 0.58 | 0.28 |
| rs6565478 | 0.25 | 0.71 | 0.21 | 0.84 | 0.25 | 0.79 | 0.23 | 0.90 | 0.27 | 0.96 | 0.37 | 0.46 | 0.65 | 0.72 | 0.50 | 0.21 | 0.56 |
| rs12603074 | 0.06 | 0.51 | 0.13 | 0.62 | 0.07 | 0.54 | 0.11 | 0.48 | 0.36 | 0.90 | **0.04** | 0.17 | 0.85 | 0.53 | 0.74 | 0.07 | 0.71 |
| rs9915378 | 0.31 | 0.61 | 0.25 | 0.70 | 0.14 | 0.76 | 0.20 | 0.26 | 0.85 | 0.75 | 0.19 | 0.19 | 0.70 | 0.59 | 0.46 | 0.14 | 0.97 |
| rs9674559 | 0.88 | 0.95 | 0.86 | 0.31 | 0.66 | 0.61 | 0.83 | 0.26 | 0.42 | 0.49 | 0.52 | 0.08 | 0.18 | 0.20 | 0.68 | 0.63 | 0.18 |
| rs4969230 | 0.23 | 0.90 | 0.06 | 0.58 | **0.04** | 0.69 | 0.06 | 0.07 | 0.19 | 0.41 | 0.33 | **0.02** | 0.52 | 0.29 | 0.31 | 0.06 | 0.28 |
| rs8078829 | 0.23 | 0.23 | 0.18 | 0.40 | 0.26 | 0.31 | 0.22 | 0.77 | **0.04** | 0.51 | 0.74 | 0.63 | 0.64 | 0.48 | 0.23 | 0.30 | 0.13 |
| rs7215564 | 0.84 | 0.38 | 0.84 | 1.00 | 0.73 | 0.94 | 0.83 | 0.94 | 0.09 | 0.60 | 0.86 | 0.20 | 0.83 | 1.00 | 0.30 | 0.75 | 0.32 |
| rs9894401 | 0.52 | 0.74 | 0.75 | 0.52 | 0.71 | 0.79 | 0.78 | 0.21 | 0.67 | 0.12 | 0.19 | 0.19 | 0.17 | 0.37 | 0.24 | 0.56 | 0.30 |
| rs7208536 | 0.78 | 0.96 | 0.84 | 0.47 | 0.90 | 0.81 | 0.91 | 0.32 | 0.80 | 0.18 | 0.42 | 0.13 | 0.18 | 0.34 | 0.35 | 0.83 | 0.37 |
| rs11150744 | **0.03** | 0.52 | 0.05 | 0.38 | **0.03** | 0.37 | 0.05 | 0.40 | **0.02** | 0.66 | **0.03** | 0.12 | 0.63 | 0.32 | 0.50 | **0.03** | 0.07 |
| rs4969266 | 0.57 | 0.62 | 0.43 | 0.37 | 0.59 | 0.36 | 0.54 | 0.26 | 0.29 | 0.52 | 0.87 | 0.24 | 0.34 | 0.24 | 0.78 | 0.63 | 0.33 |
| rs4969429 | 0.60 | 0.79 | 0.91 | 0.82 | 0.90 | 0.76 | 0.93 | 0.30 | 0.82 | 0.31 | 0.52 | 0.50 | 0.97 | 0.87 | 0.60 | 0.93 | 0.99 |
| rs7219896 | 0.08 | 0.38 | 0.27 | 0.36 | 0.33 | 0.27 | 0.29 | 0.74 | 0.07 | 0.72 | 0.25 | 0.97 | 1.00 | 0.45 | 0.32 | 0.22 | 0.49 |
| rs7225574 | **0.02** | 0.78 | 0.37 | 0.31 | 0.25 | 0.45 | 0.31 | 0.35 | 0.11 | 0.20 | **0.01** | 0.26 | 0.74 | 0.26 | 0.44 | 0.12 | 0.75 |
| rs11651724 | 0.15 | 0.65 | 0.53 | 0.40 | 0.47 | 0.46 | 0.49 | 0.73 | 0.89 | 0.26 | 0.17 | 0.81 | 0.80 | 0.35 | 0.87 | 0.38 | 0.91 |
| rs4969444 | 0.07 | 0.17 | 0.23 | 0.23 | 0.27 | 0.15 | 0.24 | 0.48 | 0.09 | 0.80 | 0.22 | 0.67 | 0.89 | 0.26 | 0.21 | 0.17 | 0.41 |
| rs2672886 | 0.16 | 0.39 | 0.12 | 0.48 | 0.11 | 0.45 | 0.12 | 0.32 | 0.90 | 0.90 | 0.22 | 0.44 | 0.98 | 0.67 | 0.23 | 0.11 | 0.97 |
| rs2333990 | 0.54 | 0.09 | 0.90 | 0.38 | 0.95 | 0.18 | 0.88 | 0.94 | 0.55 | 0.81 | 0.49 | 0.44 | 0.71 | 0.60 | 0.28 | 0.96 | 0.77 |
| rs2138125 | **0.03** | 0.22 | **0.01** | 0.61 | **0.01** | 0.50 | **0.01** | 0.48 | 0.19 | 0.86 | 0.07 | 0.25 | 0.31 | 0.86 | 0.22 | **0.01** | 0.32 |
| rs2048753 | **0.04** | 0.68 | 0.11 | 0.69 | 0.05 | 0.77 | 0.10 | 0.31 | 0.77 | 0.86 | **<0.01** | 0.69 | 0.87 | 0.76 | 0.60 | 0.05 | 0.95 |
| rs2589133 | 0.19 | 0.12 | 0.11 | 0.23 | 0.05 | 0.21 | 0.09 | 0.26 | 0.28 | 0.97 | **0.01** | 0.34 | 0.26 | 0.53 | 0.21 | 0.06 | 0.29 |
| rs7221948 | 0.85 | 0.63 | 0.98 | 0.76 | 0.98 | 0.73 | 0.98 | 0.84 | 0.20 | 0.79 | 0.96 | 0.52 | 0.36 | 0.83 | 0.60 | 0.99 | 0.26 |
| rs7217174 | 0.88 | 0.16 | 0.65 | 0.56 | 0.89 | 0.33 | 0.70 | 0.78 | 0.65 | 0.13 | 0.55 | 0.20 | 0.75 | 0.76 | 0.08 | 0.92 | 0.51 |
| rs2672901 | 0.88 | 0.11 | 0.25 | 0.27 | 0.12 | 0.16 | 0.21 | 0.34 | 0.40 | 0.93 | 0.43 | 0.50 | 0.22 | 0.38 | 0.20 | 0.19 | 0.32 |
| rs7215379 | 0.69 | 0.72 | 0.52 | 0.56 | 0.13 | 0.59 | 0.43 | 0.66 | 0.38 | 0.57 | 0.26 | 0.31 | 0.53 | 0.40 | 0.72 | 0.16 | 0.46 |
| rs2589143 | 0.88 | 0.17 | 0.17 | 0.34 | 0.21 | 0.22 | 0.19 | 0.64 | 0.22 | 0.65 | 0.66 | 0.65 | 0.18 | 0.31 | 0.44 | 0.25 | 0.21 |
| rs746405 | 0.80 | 0.21 | 0.21 | 0.37 | 0.25 | 0.29 | 0.19 | 0.23 | 0.39 | 0.27 | 0.68 | 0.74 | 0.28 | 0.25 | 0.93 | 0.31 | 0.39 |
| rs7219745 | **0.03** | 0.17 | **0.01** | 0.60 | **0.01** | 0.42 | **0.01** | 0.21 | 0.66 | 0.19 | **0.03** | 0.34 | 0.84 | 0.83 | 0.09 | **0.01** | 0.74 |
| rs2589158 | 0.84 | 0.58 | 0.88 | 0.18 | 0.81 | 0.24 | 0.85 | 0.38 | 0.81 | 0.22 | 0.90 | 0.72 | 0.88 | 0.08 | 0.90 | 0.81 | 0.89 |
| rs4969219 | 0.97 | 0.20 | 0.43 | 0.24 | 0.28 | 0.20 | 0.37 | 0.60 | 0.59 | 0.18 | 0.71 | 0.75 | 0.26 | 0.15 | 0.57 | 0.39 | 0.46 |
| rs3829572 | 0.81 | 0.29 | 0.17 | 0.44 | 0.25 | 0.36 | 0.17 | 0.46 | 0.37 | 0.35 | 0.59 | 0.96 | 0.66 | 0.36 | 0.55 | 0.28 | 0.45 |
| rs2589155 | 0.06 | 0.18 | **0.02** | 0.62 | **0.02** | 0.48 | **0.02** | 0.32 | 0.97 | 0.38 | 0.05 | 0.49 | 0.54 | 0.85 | 0.16 | **0.02** | 1.00 |
| rs2589150 | 0.23 | 0.52 | 0.09 | 0.93 | 0.05 | 0.79 | 0.08 | 0.16 | 0.96 | 0.64 | 0.29 | 0.81 | 0.63 | 0.98 | 0.40 | 0.07 | 0.99 |
| rs2589149 | 0.96 | 0.76 | 0.99 | 0.48 | 0.99 | 0.52 | 0.99 | 0.30 | 0.71 | 0.54 | 0.90 | 0.90 | 0.66 | 0.41 | 0.99 | 0.99 | 0.69 |
| rs2672893 | 0.05 | 0.32 | **0.02** | 0.83 | **0.02** | 0.75 | **0.02** | 0.36 | 0.48 | 0.48 | 0.06 | 0.56 | 0.73 | 0.87 | 0.32 | **0.03** | 0.61 |
| rs12943041 | 0.90 | 0.69 | 0.50 | 0.56 | 0.40 | 0.59 | 0.44 | 0.36 | 0.90 | 0.72 | 0.73 | 0.66 | 0.68 | 0.46 | 0.66 | 0.44 | 0.67 |
| rs2672890 | 0.84 | 0.35 | 0.74 | 0.82 | 0.82 | 0.58 | 0.74 | 0.82 | 0.55 | 0.66 | 0.76 | 0.30 | 0.88 | 0.71 | 0.47 | 0.84 | 0.75 |
| rs2589118 | 0.44 | 0.47 | 0.27 | 0.85 | 0.22 | 0.80 | 0.26 | 0.86 | 0.87 | 0.37 | 0.59 | 0.51 | 0.93 | 0.85 | 0.50 | 0.32 | 0.98 |
| rs9912092 | 0.23 | 0.67 | 0.42 | 0.79 | 0.33 | 0.83 | 0.39 | 0.23 | 0.51 | 0.34 | 0.26 | 0.66 | 0.33 | 0.62 | 0.34 | 0.36 | 0.39 |
| rs2589142 | 0.77 | 0.79 | 0.73 | 0.71 | 0.96 | 0.83 | 0.82 | 0.62 | 0.82 | 0.35 | 0.54 | 0.91 | 0.97 | 0.73 | 0.86 | 0.98 | 0.90 |
| rs6565484 | 0.31 | 0.58 | 0.34 | 0.78 | 0.24 | 0.78 | 0.32 | 0.23 | 0.31 | 0.50 | 0.47 | 0.93 | 0.27 | 0.96 | 0.34 | 0.31 | 0.33 |
| rs2289762 | 0.10 | 0.60 | 0.21 | 0.85 | 0.14 | 0.79 | 0.21 | 0.11 | 0.87 | 0.50 | 0.14 | 0.92 | 0.73 | 0.88 | 0.28 | 0.14 | 0.65 |
| rs7219553 | 0.40 | 0.33 | 0.51 | 0.36 | 0.41 | 0.32 | 0.40 | 0.42 | 0.77 | 0.77 | 0.78 | 0.75 | 0.74 | 0.48 | 0.25 | 0.55 | 0.79 |
| rs2289766 | **0.04** | 0.33 | 0.29 | 0.65 | 0.31 | 0.41 | 0.24 | 0.86 | 0.29 | 0.10 | 0.07 | 0.50 | 0.33 | 0.45 | 0.56 | 0.13 | 0.95 |
| rs2280146 | 0.17 | 0.30 | 0.30 | 0.67 | 0.39 | 0.35 | 0.29 | 0.95 | 0.05 | 0.41 | 0.25 | 0.90 | 0.89 | 0.57 | 0.71 | 0.23 | 0.30 |
| rs9898178 | 0.75 | 0.54 | 0.61 | 0.45 | 0.26 | 0.52 | 0.55 | 0.68 | 0.19 | 0.91 | 0.60 | 0.80 | 0.79 | 0.56 | 0.86 | 0.61 | 0.35 |
| rs12951596 | 0.86 | 0.61 | 0.77 | 0.75 | 0.45 | 0.76 | 0.73 | 0.56 | 0.14 | 0.84 | 0.58 | 0.86 | 0.98 | 0.85 | 0.81 | 0.75 | 0.43 |
| rs1468030 | 0.10 | 0.08 | 0.18 | 0.31 | 0.13 | 0.18 | 0.17 | 0.06 | 0.38 | 0.21 | 0.40 | 0.50 | 0.74 | 0.46 | 0.07 | 0.21 | 0.66 |
| rs7220348 | 0.33 | **0.01** | 0.09 | 0.25 | 0.21 | **0.03** | 0.13 | 0.15 | 0.26 | 0.18 | 0.48 | 0.56 | 0.69 | 0.56 | **0.01** | 0.35 | 0.69 |
| rs9897968 | 0.38 | 0.59 | 0.96 | 0.85 | 0.91 | 0.70 | 0.92 | 0.54 | 0.60 | 0.92 | 0.33 | 0.92 | 0.86 | 0.80 | 0.65 | 0.66 | 0.94 |
| rs9901846 | 0.77 | 0.49 | 0.44 | 0.46 | 0.54 | 0.45 | 0.51 | 0.91 | 0.23 | 0.26 | 0.73 | 0.73 | 0.64 | 0.41 | 0.52 | 0.74 | 0.94 |
| rs9908270 | 0.91 | 0.29 | 0.65 | 0.30 | 0.83 | 0.17 | 0.75 | 0.56 | 0.21 | 0.36 | 0.60 | 0.97 | 0.48 | 0.44 | 0.52 | 0.94 | 0.95 |
| rs2271602 | 0.23 | 0.15 | 0.24 | 0.33 | 0.27 | 0.23 | 0.25 | 0.30 | 0.18 | 0.13 | 0.37 | 0.87 | 0.99 | 0.47 | 0.07 | 0.32 | 0.59 |
| rs2271608 | 0.29 | 0.39 | 0.83 | 0.63 | 0.74 | 0.51 | 0.76 | 0.12 | 0.33 | 0.28 | 0.31 | 0.31 | 0.62 | 0.67 | 0.18 | 0.54 | 0.78 |
| rs4969227 | 0.46 | **0.02** | 0.05 | 0.85 | 0.64 | 0.48 | 0.10 | 0.65 | 0.80 | **0.01** | 0.83 | 0.75 | 0.35 | 0.84 | **0.02** | 0.56 | 0.36 |
| rs11655629 | 0.57 | 0.96 | 0.67 | 1.00 | 0.70 | 0.99 | 0.71 | 0.07 | **0.03** | 0.37 | 0.63 | 0.31 | 0.13 | 0.73 | 0.61 | 0.73 | 0.07 |
| rs1877926 | 0.25 | 0.25 | 0.47 | 0.61 | 0.46 | 0.42 | 0.47 | 0.29 | 0.92 | 0.13 | 0.42 | 0.71 | 0.78 | 0.82 | 0.17 | 0.48 | 0.90 |
| rs9911223 | 0.94 | 0.46 | 0.55 | 0.93 | 0.92 | 0.67 | 0.66 | 0.29 | 0.83 | 0.71 | 0.82 | 0.56 | 0.45 | 0.78 | 0.94 | 0.91 | 0.82 |
| rs7219318 | 0.86 | 0.29 | 0.76 | 0.75 | 0.85 | 0.68 | 0.81 | 0.11 | 0.16 | 0.67 | 0.55 | 0.63 | 0.67 | 0.65 | 0.66 | 0.86 | 0.59 |
| rs6565498 | 0.33 | 0.53 | 0.50 | 0.83 | 0.79 | 0.53 | 0.51 | 0.31 | 0.06 | 0.77 | 0.58 | 0.83 | 0.91 | 0.86 | 0.52 | 0.51 | 0.29 |
| rs2878052 | 0.24 | 0.13 | 0.49 | 0.32 | 0.47 | 0.21 | 0.48 | 0.15 | 0.21 | 0.70 | 0.23 | 0.59 | 0.76 | 0.56 | 0.11 | 0.39 | 0.45 |
| rs2271612 | 0.25 | **<0.01** | 0.20 | 0.08 | 0.41 | **<0.01** | 0.24 | 0.55 | 0.91 | 0.36 | 0.56 | 0.88 | 0.73 | 0.17 | **0.01** | 0.30 | 0.90 |
| rs7209380 | 0.18 | 0.15 | 0.31 | 0.09 | 0.09 | 0.08 | 0.18 | 0.05 | 0.42 | 0.82 | 0.05 | 0.87 | 0.24 | 0.22 | 0.10 | 0.10 | 0.26 |
| rs9907231 | 0.68 | 0.73 | 0.74 | 0.34 | 0.78 | 0.60 | 0.80 | **0.03** | 0.28 | 0.08 | 0.72 | 0.08 | 0.11 | 0.10 | 0.17 | 0.79 | 0.16 |
| rs6420481 | 0.83 | **0.02** | 0.19 | 0.55 | 0.90 | 0.43 | 0.48 | 0.47 | 0.80 | 0.25 | 0.33 | 0.26 | 0.08 | 0.54 | 0.20 | 0.88 | 0.21 |
| rs1468027 | 0.07 | 0.33 | 0.12 | 0.58 | 0.13 | 0.41 | 0.12 | 0.77 | 0.79 | 0.47 | 0.05 | 0.22 | 0.36 | 0.51 | 0.37 | 0.10 | 0.65 |
| rs7225525 | 0.11 | 0.41 | 0.68 | 0.82 | 0.74 | 0.88 | 0.81 | 0.84 | 0.80 | 0.33 | 0.08 | 0.08 | 0.18 | 0.82 | 0.25 | 0.55 | 0.47 |
| rs7224748 | 0.61 | 0.10 | 0.53 | 0.37 | 0.56 | 0.76 | 0.87 | 0.39 | 0.28 | 0.40 | 0.09 | 0.15 | **0.01** | 0.41 | 0.39 | 0.51 | **0.04** |
| rs9908043 | 0.18 | 0.62 | 0.21 | 0.91 | 0.77 | 0.77 | 0.30 | 0.89 | 0.05 | 0.47 | 0.35 | 0.87 | 0.65 | 0.95 | 0.43 | 0.80 | 0.25 |
| rs3751934 | 0.47 | 0.77 | 0.41 | 0.18 | 0.86 | 0.36 | 0.59 | 0.77 | 0.39 | 0.25 | 0.80 | 0.65 | 0.11 | 0.20 | 0.99 | 0.83 | 0.17 |
| rs3751932 | 0.68 | 0.55 | 0.34 | 0.82 | 0.72 | 0.97 | 0.50 | **0.02** | 0.06 | 0.08 | 0.72 | 0.18 | 0.19 | 0.59 | 0.16 | 0.69 | 0.06 |
| rs1062935 | 0.76 | 0.50 | 0.41 | 0.92 | 0.77 | 0.86 | 0.51 | 0.90 | 0.59 | 0.60 | 0.50 | 0.36 | 0.99 | 0.92 | 0.46 | 0.86 | 0.98 |
| rs6565507 | 0.47 | 0.36 | 0.22 | 0.73 | 0.73 | 0.71 | 0.37 | 0.69 | **0.04** | 0.06 | 0.34 | 0.31 | 0.23 | 0.73 | 0.23 | 0.59 | 0.05 |
| rs7502124 | 0.76 | 0.22 | 0.26 | 0.61 | 0.80 | 0.34 | 0.46 | 0.21 | **0.04** | 0.10 | 0.32 | 0.26 | 0.15 | 0.61 | 0.22 | 0.83 | **0.04** |
| rs6565508 | 0.71 | 0.45 | 0.47 | 0.92 | 0.68 | 0.91 | 0.71 | 0.33 | 0.47 | 0.54 | 0.31 | 0.93 | 0.66 | 0.95 | 0.55 | 0.73 | 0.46 |
| rs11653897 | 0.51 | 0.37 | 0.31 | **0.04** | 0.59 | 0.07 | 0.43 | 0.45 | 0.27 | 0.14 | 0.49 | 0.05 | **0.04** | 0.05 | 0.74 | 0.63 | 0.07 |
| rs1399571 | 0.63 | 0.19 | 0.58 | 0.41 | 0.58 | 0.21 | 0.75 | 0.49 | 0.96 | 0.69 | 0.12 | 0.49 | 0.72 | 0.29 | 0.67 | 0.60 | 0.75 |
| rs6565511 | 0.26 | 0.89 | 0.70 | 0.99 | 0.97 | 0.96 | 0.92 | 0.16 | **0.01** | **0.01** | 0.92 | 0.69 | 0.16 | 0.92 | 0.30 | 0.96 | **0.01** |
| rs7219486 | 0.67 | 0.45 | 0.69 | 0.78 | 0.75 | 0.57 | 0.90 | 0.40 | 0.06 | 0.32 | 0.18 | 0.99 | 0.18 | 0.75 | 0.65 | 0.83 | 0.07 |
| rs4969331 | 0.75 | 0.92 | 0.30 | 0.16 | 0.65 | 0.38 | 0.45 | 0.32 | 0.44 | **0.01** | 0.37 | 0.09 | **0.04** | 0.09 | 0.26 | 0.85 | 0.05 |
| rs8081168 | 0.29 | 0.27 | 0.61 | 0.07 | 0.25 | 0.14 | 0.45 | 0.31 | 0.58 | 0.13 | 0.12 | 0.63 | 0.16 | 0.14 | 0.55 | 0.24 | 0.27 |
| rs7219221 | 0.11 | 0.25 | 0.16 | 0.58 | 0.17 | 0.43 | 0.17 | 0.06 | 0.14 | 0.73 | 0.28 | 0.33 | 0.88 | 0.98 | 0.12 | 0.15 | 0.34 |
| rs7225916 | 0.29 | 0.66 | 0.53 | 0.70 | 0.63 | 0.66 | 0.58 | 0.61 | 0.53 | 0.39 | 0.41 | 0.90 | 0.68 | 0.66 | 0.53 | 0.54 | 0.71 |
| rs7502321 | 0.44 | 0.89 | 0.62 | 0.94 | 0.89 | 0.93 | 0.77 | 0.48 | 0.96 | 0.99 | 0.64 | 0.68 | 0.96 | 0.96 | 0.99 | 0.76 | 0.98 |
| rs8072124 | 0.21 | 0.41 | 0.31 | 0.83 | 0.27 | 0.73 | 0.31 | 0.29 | 0.78 | 0.77 | 0.12 | 0.25 | 0.73 | 0.87 | 0.28 | 0.25 | 0.74 |
| rs4969349 | 0.65 | 0.29 | 0.34 | 0.26 | 0.16 | 0.23 | 0.22 | 0.65 | 0.06 | 0.44 | 0.20 | 0.49 | 0.05 | 0.21 | 0.75 | 0.18 | 0.10 |
| rs4969355 | 0.52 | 0.11 | 0.30 | 0.23 | 0.29 | 0.16 | 0.29 | 0.38 | 0.18 | 0.21 | 0.54 | 0.48 | 0.70 | 0.34 | 0.10 | 0.47 | 0.45 |
| rs9906253 | 0.96 | 0.13 | 0.22 | 0.15 | 0.16 | 0.14 | 0.19 | 0.76 | 0.19 | 0.87 | 0.80 | 0.78 | 0.50 | 0.22 | 0.30 | 0.29 | 0.40 |
| rs12051877 | 0.14 | 0.25 | **0.04** | 0.59 | 0.34 | 0.39 | 0.07 | 0.06 | 0.91 | **<0.01** | 0.74 | 0.15 | 0.33 | 0.80 | **<0.01** | 0.35 | 0.28 |
| rs11869351 | 0.05 | 0.05 | 0.05 | 0.47 | 0.07 | 0.23 | 0.05 | 0.46 | 0.87 | 0.27 | 0.09 | 0.08 | 0.85 | 0.67 | 0.05 | 0.06 | 0.93 |
| rs8079626 | 0.90 | 0.19 | 0.41 | 0.32 | 0.33 | 0.29 | 0.38 | 0.51 | 0.25 | 0.72 | 0.65 | 0.94 | 0.05 | 0.46 | 0.19 | 0.38 | 0.12 |
| rs12945231 | 0.91 | 0.46 | 0.72 | 0.59 | 0.90 | 0.42 | 0.76 | 0.29 | 0.39 | 0.93 | 0.91 | 0.61 | 0.87 | 0.43 | 0.88 | 0.91 | 0.73 |
| rs4969367 | **0.04** | 0.68 | 0.08 | 0.94 | 0.08 | 0.88 | 0.07 | 0.33 | 0.14 | 0.37 | 0.08 | **0.04** | 0.78 | 0.65 | 0.29 | 0.07 | 0.37 |
| rs9901648 | **0.03** | **0.03** | **0.04** | **0.02** | **0.01** | **0.02** | **0.03** | 0.63 | 0.35 | 0.94 | **0.01** | 0.59 | 0.13 | **0.04** | 0.06 | **0.02** | 0.25 |
