# Supplementary material for: Allele-Specific Down-Regulation of RPTOR Expression Induced by Retinoids Contributes to Climate Adaptations
Source: PLoS Genet. 2010 Oct 28;6(10):e1001178. doi: 10.1371/journal.pgen.1001178 (PMC2965758; doi:10.1371/journal.pgen.1001178)
Supplement: Table S4 — Population index for the resequenced regions in YRI (A), CEU (B), and the ASN (C) populations. (0.08 MB DOC) [file pgen.1001178.s008.doc]

Table S4: Population index for the resequenced regions in YRI (A), CEU (B) and the ASN (C) populations.

A

|  | ~43KB upstream region | rs11868112 nearby region | Promoter region | intron | total non-coding | coding region |
| --- | --- | --- | --- | --- | --- | --- |
| Length (Kb) | 1.4 | 2.6 | 3.2 | 21.3 | 28.4 | 6.8 |
| S | 4 | 3 | 9 | 97 | 113 | 24 |
| π (per kb) | 0.000783 | 0.000144 | 0.000862 | 0.001174 | 0.001026 | 0.000898 |
| θ_W_ (per kb) | 0.000734 | 0.000287 | 0.000704 | 0.001202 | 0.00104 | 0.001061 |
| Tajima’s D | 0.1688 | -1.1504 | 0.6877 | -0.089 | -0.0519 | -0.6247 |
| Fu & Li’s D* | 0.0259 | -1.5078 | 0.1817 | -0.044 | -0.0958 | -0.4715 |
| No. of Haplotypes | 6 | 4 | 12 | 32 | 32 | 15 |
| Haplotype Diversity | 0.7137 | 0.3327 | 0.8508 | 1 | 1 | 0.9917 |

B

|  | ~43KB upstream region | rs11868112 nearby region | Promoter region | intron | total non-coding | coding region |
| --- | --- | --- | --- | --- | --- | --- |
| Length (Kb) | 1.4 | 2.6 | 3.2 | 21.3 | 28.4 | 6.8 |
| S | 2 | 2 | 8 | 79 | 91 | 23 |
| π (per kb) | 0.000421 | 0.00033 | 0.000654 | 0.001081 | 0.000933 | 0.00111 |
| θ_W_ (per kb) | 0.000367 | 0.000191 | 0.000626 | 0.000945 | 0.000813 | 0.001017 |
| Tajima’s D | 0.2974 | 1.4592 | 0.1336 | 0.5404 | 0.5581 | 0.3727 |
| Fu & Li’s D* | 0.7979 | 0.7979 | -0.6117 | 0.623 | 0.553 | 0.2988 |
| No. of Haplotypes | 3 | 4 | 10 | 32 | 32 | 16 |
| Haplotype Diversity | 0.4919 | 0.6552 | 0.881 | 1 | 1 | 1 |

C

|  | ~43KB upstream region | rs11868112 nearby region | Promoter region | intron | total non-coding | coding region |
| --- | --- | --- | --- | --- | --- | --- |
| Length (Kb) | 1.4 | 2.6 | 3.2 | 21.3 | 28.4 | 6.8 |
| S | 3 | 5 | 8 | 71 | 87 | 22 |
| π | 0.000557 | 0.000425 | 0.000705 | 0.001031 | 0.000917 | 0.001087 |
| θ_W_ | 0.00055 | 0.000479 | 0.000626 | 0.000852 | 0.000778 | 0.000972 |
| Tajima’s D | 0.0283 | -0.3014 | 0.3758 | 0.7902 | 0.6726 | 0.4757 |
| Fu & Li’s D* | -0.283 | -1.5255 | -1.2559 | 0.9864 | 0.4901 | 0.0161 |
| No. of Haplotypes | 4 | 7 | 10 | 32 | 32 | 16 |
| Haplotype Diversity | 0.623 | 0.7238 | 0.881 | 1 | 1 | 1 |
