# Supplementary material for: Allele-Specific Down-Regulation of RPTOR Expression Induced by Retinoids Contributes to Climate Adaptations
Source: PLoS Genet. 2010 Oct 28;6(10):e1001178. doi: 10.1371/journal.pgen.1001178 (PMC2965758; doi:10.1371/journal.pgen.1001178)
Supplement: Table S5 — Primers for RPTOR PCR and resequencing in this study. (0.05 MB DOC) [file pgen.1001178.s009.doc]

Table S5. Primers for *RPTOR* PCR and resequencing in this study.

| **region** | **PCR primer (5’-3’)** | **Annealing temperature (˚C)** | **Sequencing primer (5’-3’)** |
| --- | --- | --- | --- |
| coding^a^ | Ttgatgggctgatgagatga  tgatggctgtgaagatccag | 61.8 | TTCACTGTAGCTCCAAACCA  CGGCCCCCACCTCCCCACTT  CCACCTCCCCACTTCCCGCTCAG  AAAGCCAAAGGTAGGTTCCAGTCT  GCTTCTTGACTTCATCCACAG |
| coding^a^ | Cctccgtcgatgaaaaactg  cttgtaggcgatgctgttga | 62 | AGAACCGAAACCCACCCGAACAGC  GTGCCAAGCGGACCTCGTGA |
| coding^a^ | Tcctgtccttcgagaccatc  gacacgacagcgaagacaga | 58 | Actctccaggtcgtgctct  AGCTCCAGCCTGACCAACGAT  AGGACTGCTCGCTTCTGCT  GTCATGACGTACCGGGAGCACA  GGACGATGCTGCTGGACACAA  ATGACGGGCTGGGCAAAATAG |
| coding^a^ | Ccgcctgctgtacatagtga  gagtctgcgcgtgttgttta | 59 | GCCCACCCTCCCCGCACCTC  GCAAACAGAACACAACCACAATGA  CCAACTGGCGGGTGTGAAGGAA  AGTGTTCTTTCCGTGTTCG |
| promoter | GCAGGAATTGGTGGCAGGTTG  TGGTTTTCCGGGGCTTAGACAC | 64.6 | GTTGTACATCAGCAGCACTTC  AAGAAGTTCATCCGGCACCAGT  TTATAGGTGAGGAAACTGAG  CAAAATGAGAATGAAGAATAC |
| promoter | TGTCTAAGCCCCGGAAAACCAT  AAACGGGCGACACGGAACCAC | 64.6 | GTTTTCTGAGCAGTTTATCCTT  GCCACGGTGCCCTGCCTACAT  AACCACGGAGGAAAGGGACAA |
| non-coding | CCCCATTCAGGAAGCAAAACC  TCGCAGACCTGGACAAGATGAGA | 61.8 | GCAATAAAGACACAGAGAAAG  GGCACTTAGCAAACTCAGATG  CTTGCTTAGGCCCATTGAG |
| non-coding | CCTGTGTTCTCGGGCTTTTTGTT  TGGCACCATGGGGGCTCAGTAA | 68.4 | GACAAAGACGAGGATAATGAT  GGGGTCCGTGTTGGGTAAGTGC  CAGTTTCTAATTATCTATGCT |
| non-coding | GATACCACATGGGCATCTCC  CACATCCATGCGTTAGCAAG | 58.4 | GAAGATTCTAGAACCTGACAC  GCCTTCCCCAGAGCACTTG |
| non-coding | CAGAGGGATTGAAGATAAGACT  TAAATGTGGCGGAGAAAGAAAAC | 58.4 | TTGCTGGGCTGGGAAGAGACT  GAGCGGCGGTCACATCCTA  GTTCCTCTCCCTTCCCACTCG |
| non-coding | TGTGTTCTCTTTGGGTTCTG  GCTAAAGCAAAATGTCCTCA | 60.7 | CGGGGCGTTGTTCAGGTG  ATTCTTGGGTGTATTAGTCA |
| non-coding | TTCGATACCAGCGTTTCTACTA  TTCACAACATATCCACGCTAAT | 58.4 | TTCTACTATTAAAGGTGGACT  ATTCATTGGTAACCCTTTCAC |
| non-coding | GATTTTTGAACATGGTAACAG  CCTCGTTCCCTTAACCCTTC | 58.7 | TTCATGACAAAAATAAACAGC  CCGTGGACGCGCTGGTGAATG |
| non-coding | Taagacggggtcttgctctg  caccaaatgagctgtggaaa | 61.8 | GAGGTGCTTGAATCTATGAG  AGGGGTGAGCCAAGTGAGGAC  ATGCTGTGAATCCTAATGAG |
| non-coding | AGGTGGTATCTCTGCTTCC  AGATCATAAATGTAAAACTGTA | 51.7 | ACTCACTGCTCGGACAATCTTT  GGTTAGGAAAATATAGACTCAT  CAGCTGGGAGGTGGGAGAG |
| non-coding | CCCCTGGGATGATTAGA  GTCAACACGCGTGCTCTTCAAT | 55.5 | TGGATGATGGAATGGTAATGG  ATGGATAATCGGGGAAGAAGC  GCCTTGGCCTCAGCAGT |
| non-coding | CGGTGCTGGGTTTGGTTTTG  GCTGGAGGTGCTGGTCTTCTG | 61.8 | AACACAACAGAAAAAGAAACC  TTTTAATTAACTGAGGCTGAT  AACCAAGATGAAATGCCTGAA |
| non-coding | GATGCTCGGGAAGGTGGAT  AAAACGGGAGCTCTATTACAA | 58.4 | TCCCTCCCTCCCTCCCTTTAC  CATTGCCCGCAGGAGACG |
| non-coding | TAAGCGGGGAGGGTGATAAT  CTTGTCGGGGATGTAGAATGG | 61.8 | TGATAATTGTGCCCATAGTCT  CTATGGGAAGGCAAGAGAAAT |
| non-coding | CGGCTGGGTTAGTGTGGAAAAA  TAAACAGCAGAACAACGGTATT | 58.4 | CACACTTTGGTCTATTGATGA  ACAAAGGAGTGAGGAGTGGT  GTCATTCTCCTTCACTTTCA |
| non-coding | CCTTGCTCTGAGTGGGGCTGTC  CCTTGTGGAAACCGAGTGATG | 68.4 | GCTGTGGCTTGAGGTTC  TGACCGAGGCCACACCAG |
| rs11868112 nearby region | TGCAAACTCATCGTCCATCTTA  GGGCCTCCTTCGTCCTCCAG | 58.4 | GCACCTGGACTTGTTTTACTGAA  AGCATTTTCTGGGTTCTTTA |
| rs11868112 nearby region | TCTCACCCCATCTCACAAATAAG  GTCACATACGCACAATCTCAAT | 58.4 | AAAGGGATGAGTGACTGGATTGG  TGGGCAGCAGGGTCAAGTA |

^a^cDNA from lymphoblastoid cell line (LCL) is used as template.
